# Functional Diversity Facilitates Stability Under Environmental Changes in an Outdoor Microalgal Cultivation System

**DOI:** 10.3389/fbioe.2021.651895

**Published:** 2021-04-22

**Authors:** Lina Mattsson, Eva Sörenson, Eric Capo, Hanna Maria Farnelid, Maurice Hirwa, Martin Olofsson, Fredrik Svensson, Elin Lindehoff, Catherine Legrand

**Affiliations:** ^1^Department of Biology and Environmental Science, Centre of Ecology and Evolution and Microbial Model Systems, Linnaeus University, Kalmar, Sweden; ^2^Department of Chemistry, Umeå University, Umeå, Sweden; ^3^Axis Communications, Lund, Sweden; ^4^BioResM, Maroc Sarl, Safi, Morocco

**Keywords:** microalgal cultivation, functional diversity, microbial consortium, sustainability, environmental changes, algal productivity, thermal regime, polyculture

## Abstract

Functionally uniform monocultures have remained the paradigm in microalgal cultivation despite the apparent challenges to avoid invasions by other microorganisms. A mixed microbial consortium approach has the potential to optimize and maintain biomass production despite of seasonal changes and to be more resilient toward contaminations. Here we present a 3-year outdoor production of mixed consortia of locally adapted microalgae and bacteria in cold temperate latitude. Microalgal consortia were cultivated in flat panel photobioreactors using brackish Baltic Sea water and CO_2_ from a cement factory (Degerhamn, Cementa AB, Heidelberg Cement Group) as a sustainable CO_2_ source. To evaluate the ability of the microbial consortia to maintain stable biomass production while exposed to seasonal changes in both light and temperature, we tracked changes in the microbial community using molecular methods (16S and 18S rDNA amplicon sequencing) and monitored the biomass production and quality (lipid, protein, and carbohydrate content) over 3 years. Despite changes in environmental conditions, the mixed consortia maintained stable biomass production by alternating between two different predominant green microalgae (*Monoraphidium* and *Mychonastes*) with complementary tolerance to temperature. The bacterial population was few taxa co-occured over time and the composition did not have any connection to the shifts in microalgal taxa. We propose that a locally adapted and mixed microalgal consortia, with complementary traits, can be useful for optimizing yield of commercial scale microalgal cultivation.

## Introduction

Biological carbon capture is one of the methods used to reduce atmospheric CO_2_ levels (IPCC, 2018). Due to the photosynthetic ability and fast turn-over rates of microalgae, large-scale microalgal cultivation systems in association with factories may be used to capture, and utilize industrial CO_2_ emissions (Moheimani, 2013; Olofsson, 2015). The microalgal biomass may in turn be used as a non-fossil based, source of raw material where lipids can be used as biofuels, proteins for animal feed, and carbohydrates for bioplastics (Chisti, 2007; Duong et al., 2012; Mathiot et al., 2019). The full potential of microalgal cultivation has not yet been realized at the commercial scale for fuel, due to limitations such as unstable productivity, grazer and pathogen contamination, and high energy requirements during harvest and extraction (Nalley et al., 2014). Economic sustainability in large scale cultivation can be reached by recycling resources for cultivation together with the use of stable microbial consortia that have a resilience toward fluctuating environmental conditions. But more studies are needed to know how cultures with microbial consortia perform in outdoor large-scale systems.

The use of monocultures has remained the paradigm since the start of commercial microalgal cultivation (Borowitzka, 1999; Kaparapu and Geddada, 2020) where *Chlorella, Spirulina*, and *Dunaliella* are common microalgae genera used. The maintenance of large-scale monocultures requires high standards regarding cleaning, as pathogens and invasions of grazers, can compromise the viability and integrity of the culture. In addition, environmental conditions need to be kept constant, which may lead to high costs in terms of both energy and labor (Cooper and Smith, 2015). In outdoor conditions, daily and seasonal ambient temperature variations can affect productivity and stability of the system (Maroubo et al., 2018; Gupta et al., 2019). Temperatures outside of the microalgal strains optimal temperature range can alter the enzymatic processes in microalgae leading to changes in biomass productivity and quality (Ras et al., 2013). Therefore, it is crucial to find optimization strategies that allow stable production. To overcome the challenges of monocultures, attempts to use genetically modified microalgae (manipulated metabolic pathways) in lab scale, have resulted in increased biomass yield and production of high value compounds such as lipids (Radakovits et al., 2010). However, the use of such organisms may, if released to the environment, cause harmful algal blooms with severe effects in the trophic chain (Flynn et al., 2013).

Several studies have assessed whether cultures with multiple algal strains may enhance biomass productivity (Ptacnik et al., 2008; Weis et al., 2008; Cardinale et al., 2011; Shurin et al., 2014; Olofsson, 2015; Beyter et al., 2016). Up to a certain diversity, combinations of microalgae seem to produce more biomass than either of them cultivated alone, an effect known as overyielding (Tilman, 1997; Loreau and Hector, 2001). The two main mechanisms proposed behind the diversity-productivity relationship are the “sampling effect” and the “complementarity effect” (Loreau and Hector, 2001; Cardinale et al., 2011). The “sampling effect” theory proposes that highly productive species, optimal for ambient conditions, are more likely to be present in a diverse community and thereby increases the overall productivity. The “complementarity effect” hypothesizes that an assortment of species can fill functional niches in a more efficient way than any species alone, leading to a more efficient uptake of nutrients and utilization of light and temperature. A mixed algal community with complementary functional traits may therefore not only increase the biomass yield but also metabolite productivity and the resilience of cultures (Corcoran and Boeing, 2012; Dae-Hyun et al., 2017; Olofsson et al., 2019) to buffer drops in productivity at fluctuating conditions (Nalley et al., 2014). This concept is of particular interest in temperate regions with large differences in light and temperature between seasons that can challenge stability. Thus, the presence of several taxa could make the system less sensitive to both seasonal and diurnal environmental fluctuations as well as contaminations (Krichen et al., 2019). However, how these theories apply to large-scale outdoor microalgal cultivation is yet unknown since most of the current knowledge regarding the performance of cultures with multiple algal strains stems from laboratory-based studies. For example, the work of Olofsson et al. (2019) showed that stability and quality of biomass was not directly related to the microalgal community composition, since production of biomass was maintained while taxonomic shifts occurred. This could be a result of high functional diversity, which is when the presence of several functional groups, rather than taxonomic groups, help to maintain the rate of system processes in response to environmental fluctuations (Carpentar et al., 2006).

Heterotrophic bacteria can also play a major role in microalgal productivity (Zhang et al., 2018). Diverse bacterial populations have been found to be abundant in small scale (5–200 mL) microalgal monocultures (Fulbright et al., 2018). The most studied example is the single-celled green algae, *Chlorella* and its associated bacteria that promote the growth of the algae by exchange of organic and inorganic carbon sources (Cho et al., 2015). Several studies have also been conducted on mixed algae-bacterial communities in wastewater systems where symbiotic relationships increased the efficiency of the water treatment (Lee et al., 2016; Chen et al., 2019). Although, studies characterizing and assessing the impact of bacteria on microalgal cultivation remain few (Beyter et al., 2016; Fulbright et al., 2018), mutualistic relationships between microalgae and bacteria are expected to be prevalent in the environment (Seymour et al., 2017). The presence of heterotrophic bacteria can increase growth rate, cell mass and lipid content in microalgal cultures (Cho et al., 2015). How these observations apply to microalgae production systems is unknown as wastewater systems differ from biomass production systems due to the presence of high amounts of inorganic and organic compounds. Thus, there are many questions yet to be answered concerning the presence and impact of bacteria on both productivity and resilience in microalgal cultivation systems.

A pilot-scale outdoor photobioreactor in close proximity to a cement factory was set up to capture part of their CO_2_ emissions (Heidelberg Cement Group, Cementa AB, Degerhamn, Sweden). The system (1600–3200 L) was run during spring, summer and fall seasons for three consecutive years with a microbial consortia originating from the Baltic Sea. In this study, we investigated the impact of seasonal and diurnal variations in light and temperature on the quality and production of microalgal biomass as well as the composition of the microbial community. This was done by monitoring the biomass yield and identification of the microbial consortia (algae and bacteria) using rDNA amplicon (16S and 18S) sequencing together with correlation network analysis. The findings of this study are important for understanding how to maintain stability of biomass production and quality over time in the context of outdoor cultivation of a microbial consortium. This can help to implement strategies to increase resilience to temperature fluctuations that make microalgal cultivation more sustainable.

## Materials and Methods

### Study Site, Outdoor Photobioreactor, and Sampling

The study was conducted in a closed photobioreactor (PBR) system, located in Degerhamn on the island of Öland (SE Sweden) (N 56° 35.2707, E 16° 40.7902) at the Cementa AB (HeidelbergCement Group) facility for cement production. This facility, was until its closure in 2019, one of the three large cement factories in the Baltic Sea region using limestone as raw material (active years: 1888–2019). The PBR–a Green Wall Panel (GWP-II) from Fotosintetica & Microbiologica s.r.1 (F&M), Florence, Italy (WO2011/013104) – was installed in 2014. It consisted of 4 low density disposable polyethylene (LDPE) cultivation chambers with 12 m steel structures, with a total culture volume of approximately 1600 L covering a land area of 50 m^2^ including piping system and electromechanical equipment. The hermetical seal together with the positive inner pressure of the cultivation chambers prevents contaminations from entering the system. In the summer of 2016 (July 2nd – July 10th), the PBR was doubled in size to 8 panels and a total of 3200 L. The PBR system was originally inoculated with a Baltic Sea microbial community that was maintained for 3 years (2014–2016) by recurrent harvests 2–3 times a week during cultivation season (approximately April–November, dependent on yearly variations in temperature and timing of maintenance stops at the cement industry) and stored during the winter (3–6 months) in laboratory containers (100–300 L) under light (16:8 L:D cycle, 16°C) and aeration. During regular operation the biomass was recovered from bulk medium with a volume renewal of 0.2–0.3 per day. Temperature, pH and dissolved oxygen were monitored continuously by logging and remote transfer of data. Nutrients and vitamins were supplied in the form of Cell-Hi f/2 powder (Varicon Aqua) to the culture on a regular basis to avoid limitation. Prefiltered (1 μm) Baltic Sea water was used as liquid medium. Light (photosynthetic active radiation; PAR) data was obtained from the Swedish Meteorological and Hydrological Institute (SMHI)^1^. Temperature was monitored in the PBR panels every minute. On the dates when temperature data was missing, a conversion of SMHI-values was done using the slope equation of the regression of SMHI temperature and PBR temperature.

Cement flue gas (12–15% CO_2_) from Cementa AB was used as a CO_2_ source (Olofsson, 2015) and directed through a pipeline from the flue stack of the cement factory and bubbled into the panels. Flue gas was introduced to the PBR during daylight to maintain the pH within given setpoints. Solenoid valves were switched at the high pH setpoint 8.0 from air to flue gas and to air again when reaching the low pH setpoint (7.5–7.8) (from April 2014 to July 2016) to keep the pH setpoint range in the system. From July 2016 and onward the flue gas was injected at pH 8.0 by intermittent pulses (on: 5–20 s, off: 20–45 s) until pH decreased to the low setpoint (7.8). During the study the pH ranged 5.5–8.4 with an average of 7.7.

Overall performance was measured as productivity and density of biomass (estimated by dry weight (DW), 2–3 times a week) and quality of biomass (lipid, carbohydrates, and proteins) at microalgal community level. For dry weight measurements microalgal culture (5–20 mL) was filtered onto a rinsed, pre- dried and weighed 47 mm GF/F (Whatman) filter and placed in an aluminum cup to dry (100°C) overnight. The following day, the filter was weighed again from which the difference in weight (microalgal dry weight) could be calculated. Although, small free-living bacteria are expected to pass through the 0.7 μm GF/F filter used in this study, larger and attached bacteria in the system may contribute to the total biomass. Biomass productivity was calculated according to Equation 1.

(1) $$((\left(\mathrm{DW}\left(g\right)t_{2}-\left(\mathrm{DW}\left(g\right)t_{1}\times \mathrm{dilution}\mathrm{factor}\right)/\left(t_{2}-t_{1}\right)\right)\times \mathrm{volume}\mathrm{PBR})/\mathrm{areaPBR}$$

In Equation (1), DW refers to the biomass density measured and t represents the specific time point at which these compounds were measured.

Filters used for the characterization of the microbial communities using DNA methods were obtained by the collection and filtration of 3–10 mL microalgal culture onto a 0.2 μm Supor filter (Pall Corporation), amended with 1 mL RNAlater (Invitrogen), and stored at −80°C, on 16 sampling occasions (picked out based on when all background data was available and flue gas was available) throughout the 3 years.

Biomass for biomass quality analysis was recovered by centrifugation (Beckman AvantiTM J-25) during the 3 years with a force of 18.600 × *g* for 20 min (4°C) followed by 23.200× *g* for 20 min (4°C). An additional washing step with 0.1 M ammonium formiate was done to remove any salts in the biomass. Biomass quality was analyzed with the Chloroform:MeOH method (total lipids), NaOH and protein assay (total proteins, Bio-Rad DC Protein Assay) and the Phenol-H_2_SO_4_ method (total carbohydrates) according to modifications by Olofsson et al. (2019) based on previous literature (Lowry et al., 1951; Dubois et al., 1956; Bligh and Dyer, 1959). Productivity of lipids, proteins and carbohydrates (g m^–2^ d^–1^) was then calculated (Equation 2).

(2) (Biomassproductivity×metabolitecontent(%))

In Equation (2), biomass productivity refers to the calculated productivity in Equation 1 and metabolite content refers to either lipids, proteins or carbohydrates% of DW.

### Molecular Methodology

#### DNA Extraction

DNA was extracted using the PowerWater DNA isolation kit (MO-BIO Laboratories Inc, Carlsbad CA, United States) following the manufacturer’s instructions with the modification that cells were lysed using Matrix E bead tubes (MPbio, Solon, OH, United States), shaken twice at 60 m s^–1^ for 40 s (Fastprep-24 5G, MP Nordic biolabs) and incubated with proteinase K (20 mg mL^–1^, total of 1% of sample) in 55°C for 1 h to digest contaminating proteins. Extracted DNA yields and purity were measured using Nanodrop 2000 (Thermo Fisher Scientific, Waltham, MA, United States).

#### PCR Amplification and Sequencing of 18S and 16S rRNA Gene Fragments

A two-step PCR procedure was done to amplify the targeted gene regions and attach handles and indexes to prepare samples. The first PCR reaction amplified a region of the 18S ribosomal RNA gene using the primers 574^∗^F (CGGTAAYTCCAGCTCYV) and 1132R (CCGTCAATTHCTTYAART) (Hugerth et al., 2014) or the 16S ribosomal RNA gene using primers 341F (CCTACGGGNGGCWGCAG) and 805R (GACTACHVGGGT ATCTAATCC) at a concentration of 0.5 μM each using KAPA master mix (0.5x stock in final volume) per reaction (KAPA Biosystems). The first PCR had an initial denaturation temperature at 98°C for 30 s followed by 20 cycles of denaturation temperature of 98°C for 10 s, annealing temperature at 50.4°C for 30 s (18S) and 58°C for 30 s (16S) and elongation at 72°C for 15 s and final elongation temperature of 72°C for 2 min (BIORAD T100 thermal cycler). The PCR amplicons were purified using AMPureXP (Beckman Coulter) and magnetic beads and rinsed twice with 80% ethanol. The quality of the amplified gene fragments was controlled using gel electrophoresis with GelRed (Biotium) as dye. The second PCR, used KAPA master mix (KAPA Biosystems) and 0.2 μM of primers i50X and i71X, where X in each case represents a specific barcode so that each sample had a unique combination of forward and reverse primers. Reaction conditions for the second PCR were an initial denaturation of 98°C for 30 s followed by 12 cycles of denaturation temperature of 98°C for 10 s, annealing at 62°C for 30 s and elongation at 72°C for 5 s with a final elongation temperature of 72°C for 2 min. The PCR amplicons were purified once more using AMPureXP (Beckman Coulter) and magnetic beads and rinsing once with 80% ethanol. Samples were pooled at equimolar concentration and sequenced 2 × 300 bp with Illumina MiSeq at SciLifeLab/NGI (Solna/Sweden).

#### Data Analysis of 16S and 18S Data

Raw sequences were quality trimmed using the dada2 pipeline in the QIIME2 software (version 2019.1) in the UPPMAX computer cluster (Uppsala, Sweden). Sequences with bases with a Phred score below 20 were deleted. Forward reads were truncated at position 290 and reverse at 210 from the 3′ end and both forward and reverse reads were trimmed from the 8th base pair in the 5′ end. The amplicon sequencing resulted in 5,442,430 18S reads, of which 67% remained after filtration, and 12,910,472 16S reads, of which 68% remained after filtration (Supplementary Tables 3, 4). Taxonomy was assigned to the sequences using the SILVA reference database based on 90% sequence similarity and amplicon sequence variants (ASVs) affiliating with chloroplast sequences were removed. Only forward reads were used for 18S rRNA gene fragments due to the long length of amplicons (Hu et al., 2016), whereas a merge was done for 16S before the taxonomic assignment. Genus identification of 18S sequences was based on the NCBI BLASTN results. In total, the dataset consisted of 3021 18S ASVs and 8690 16S ASVs. Data was rarefied to the size of the smallest library (95341 for the 18S library and 21415 for the 16S library) to perform network analyses. Because of the variation in the 16S libraries, rarefaction curves were done for the 16S libraries to assure that sequencing depth was sufficient (Supplementary Figure 4). The similarity between samples in terms of number of shared bacterial ASVs was calculated according to the Sorensen similarity coefficient (Booth et al., 2011). For PCA analysis, a centered log ratio (clr) transformation was done to remove NAs (Gloor et al., 2017) in non-rarefied data.

#### Data Availability

All sequence data was deposited in the European Nucleotide Archive. The accession number for the data is: PRJEB36394/ERP119583.

### Network Analysis

In order to investigate potential interactions between microalgae and bacteria with regard to seasonal variations in temperature and light, and therefore the effects on the production of microalgal biomass, we performed a co-occurrence analysis. In the network-based analysis, Pearson correlations were calculated from the relative abundances of both microalgal and bacterial ASVs with parameters affecting the microalgal reactor: temperature in the PBR (average, min and max), diurnal shifts in PBR temperature, light intensity (PAR), and measurements made of the biomass: density measured as dry weight (g L^–1^), and productivity (g m^–2^ day^–1^). We applied the procedure from the R package WGCNA v1.68 (Weighted Correlation Network Analysis) (Langfelder and Horvath, 2012), following guidelines from Capo et al. (2017) with slight modifications. To reduce complexity of the network, ASVs with less than 0.1% counts per library were excluded, resulting in the selection of 225 18S ASVs and 411 16S ASVs. The relative abundances of the ASVs were standardized with Hellinger transformation (function *decostand*) (Oksanen et al., 2007). For network construction, a power value of 7 was used as threshold value. A signed network of clustered ASVs was created using function *adjacency* and a minimum of 8 nodes (ASVs) per module were chosen.

#### Statistics

**FIGURE 1:**
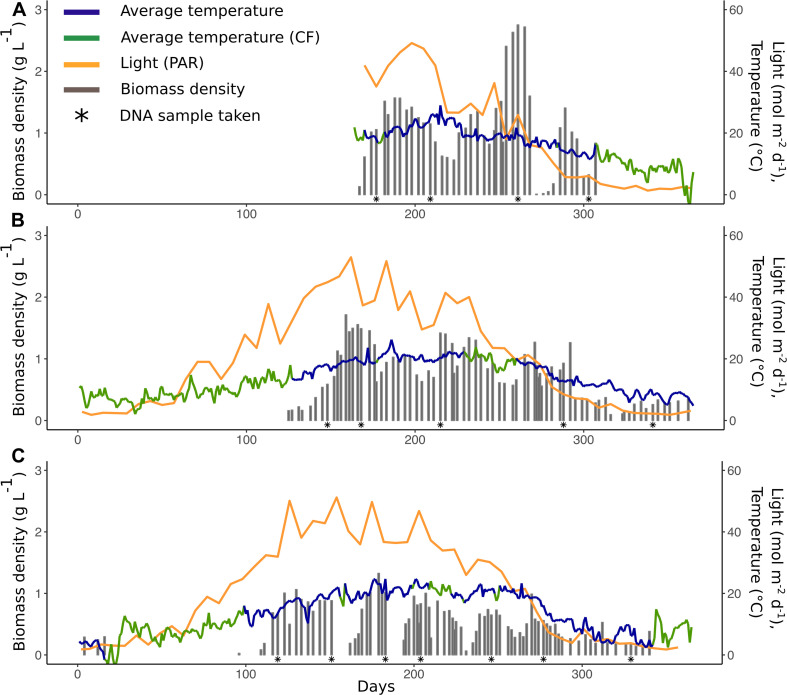
Reactor parameters for 2014 **(A)**, 2015 **(B)**, and 2016 **(C)**: Average temperature (blue line,°C), average temperature (green line,°C, calculated with conversion factor, CF), light/PAR (yellow line, mol m^–2^ d^–1^), and biomass density (gray bars, g L^–1^). Asterisks (^∗^) represent dates where samples for the community composition analysis were taken.

All statistical analyses were done in R studio (version 1.1.423). The biomass density (DW) and productivity data did not show equal variances between the 3 years and hence a non-parametric Kruskal Wallis test followed by a pairwise Wilcoxon (BH/fdr method) was performed. For all 3 years, data was obtained for the period 16th of June to 3rd of November were included in the analysis of biomass density and 19th of June to 3rd of November for productivity. A multiple linear regression was done to determine explanatory factors of biomass density and productivity. For the molecular sampling dates (16 occasions in total; Figure 1), averages for two weeks before the sampling dates were calculated for the environmental data. For biomass quality data (lipid, protein and carbohydrate content, and productivity), principal component values were extracted to perform regressions to evaluate which environmental parameters that could be coupled to the positions of the data points on that principal component axis (Göthe et al., 2019). Due to collinearity between light and temperature (Pearson’s correlation, *r* = 0.73, *t* = 25.9, *p* < 0.001), these parameters impact on the biomass density, producitivy and quality was assessed individually in the statistical analyses.

## Results

### Biomass Density and Productivity

The biomass production of the PBR was monitored for the duration of 3 years (2014–2016). The system was exposed to changes in light (0.4–59 mol m^–2^ d^–1^) and temperature (1–29°C) (Figure 1). When the system was started up in spring of each year, there was a start-up phase of lower production after which the production was maintained until the system was closed down during late fall/early winter. The environmental conditions varied between years and seasons (Figure 1 and Supplementary Table 2). Temperature also showed daily variations, diurnal shifts in temperature, that differed in magnitude between seasons (Supplementary Table 2). The biomass density, measured in dry weight (DW) g L^–1^, was on average 0.7 ± 0.5 throughout the 3 years and varied significantly between years (Kruskal-Wallis chi-squared = 31.275, *p* = 1.6 × 10^–7^) (Figure 1). The biomass density was not significantly different between the first 2 years (Pairwise Wilcoxon test, *p* > 0.05) but 2016, had 46% lower biomass density than in 2014 (Pairwise Wilcoxon test, *p* = 6.4 × 10^–6^) and 33% lower biomass density than in 2015 (Pairwise Wilcoxon test, *p* = 6 × 10^–6^).

Average temperature and light explained 23% of the variation in biomass density (Multiple linear regression, *R*^2^ = 0.23, *p* < 0.001), where temperature was the strongest driving factor. In general, biomass density was highest when there were high levels of both light and temperature, with peaks during late spring/early summer (day ∼150–200) and late summer/early fall (day ∼200–270) (Figure 1). Biomass productivity was on average 3.5 ± 3 g m^–2^ d^–1^ and did, contrary to biomass density, not differ between the years (Kruskal-Wallis chi-squared = 4.31 *p* >0.05) but 14% of the variation in productivity could be explained by temperature (Linear regression, *R*^2^ = 0.14, *p* < 0.001).

### Biomass Quality (Lipid, Protein and Carbohydrate Content)

The biochemical metabolites consisted of 19–41% lipids, 11–44% proteins and 9–37% carbohydrates (total of ash free biomass) (Supplementary Table 1). The productivity of the different metabolites was −0.18–5.6 g m^–2^ day^–1^ for lipids, −0.19–4.5 g m^–2^ day^–1^ for proteins and −0.08–4.0 g m^–2^ day^–1^ for carbohydrates (Supplementary Table 1). According to the principal component analysis (PCA), principal component PC1 explained 55% of the variation in lipid, protein and carbohydrate content (% of DW) and PC2 34% of the content (% of DW) (Supplementary Figure 1A). The variation in biomass metabolites (lipid, protein and carbohydrate % of DW) was mostly influenced by light (Linear regression, *R*^2^ = 0.45, *p* < 0.001) followed by temperature (Linear regression, *R*^2^ = 0.19, *p* < 0.001) and diurnal shifts in temperature (Linear regression, *R*^2^ = 0.09, *p* < 0.05).

According to the principal component analysis (PCA), principal component PC1 could explain 89% of the variation in lipid, protein and carbohydrate productivity and PC2 8% (Supplementary Figure 1B). The variation in biomass metabolites (lipid, protein and carbohydrate) productivity (g m^–2^ d^–1^) was mostly influenced by temperature (Linear regression, *R*^2^ = 0.19, *p* < 0.001) followed by light (Linear regression, *R*^2^ = 0.14, *p* < 0.01) and diurnal shifts in temperature (Linear regression, *R*^2^ = 0.14, *p* < 0.01).

### PBR Microbial Community Composition

**FIGURE 2:**
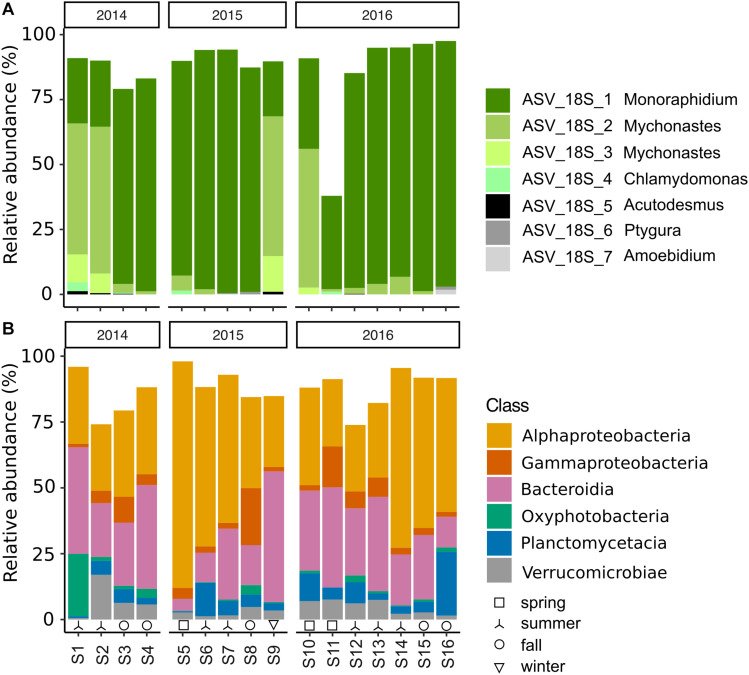
Reactor community composition of **(A)** algae (18S), **(B)** bacteria (16S) in relative abundance (%), during 2014–2016. Top 7 algal ASVs with taxonomic assignment at genus level. Bacterial ASVs grouped by class with relative abundances >1% in more than 50% of the samples are included. Seasons are indicated at the bottom: spring (□), summer (⅄), fall (∘), and winter (▽).

The microalgal community, as described from 18S rRNA gene amplicon libraries, was dominated by four ASVs throughout the 3 years, of which all were assigned to the phylum Chlorophyta (82–96%). Of these, ASV_18S_1, assigned to the genus *Monoraphidium*, was predominant in 10 of the 16 samples (relative abundance >81–95%) (Figure 2A and Supplementary Figure 3). On the other occasions, ASV_18S_2 & 3, assigned to the genus *Mychonastes*, were predominant in the microalgal community (55–68%). Additionally, ASV_18S_4, assigned to *Chlamydomonas*, was present albeit at comparably low relative abundances on two dates. According to principal component analysis (PCA), the shifts in microalgal taxa could not be explained by meteorological season (Supplementary Figure 2A).

**FIGURE 3:**
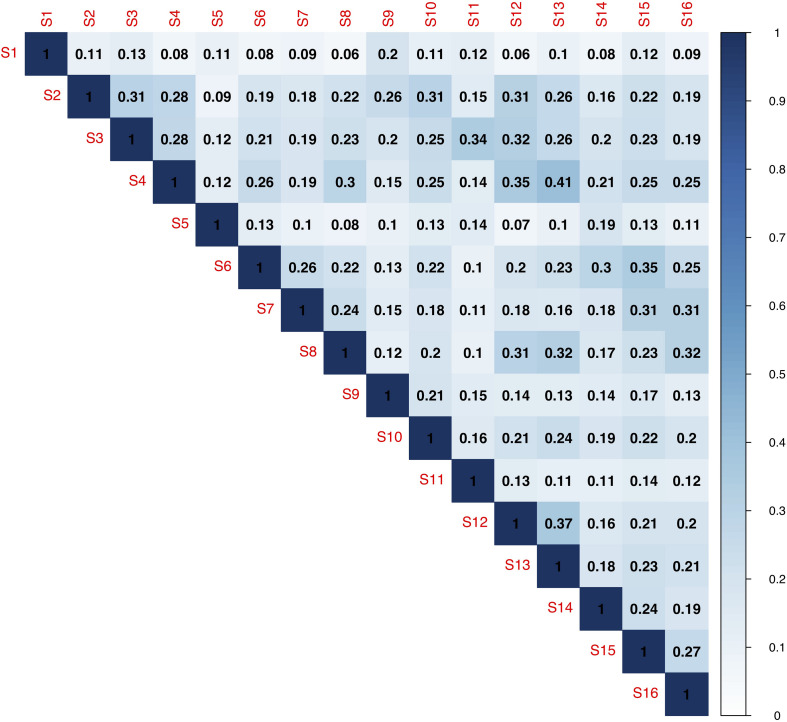
Similarity between the 16 samples in terms of composition of the bacterial communities (presence or absence of 16S ASVs) based on the Sorensen similarity coefficient.

The bacterial community was dominated by members of Proteobacteria (31–89%)–mainly Alphaproteobacteria (25–86%) and Gammaproteobacteria (1–22%)–Bacteroidia (5–50%), along with members from Planctomycetacia (0.4–24%), Verrucomicrobiae (0.5–17%), and Oxyphotobacteria (Cyanobacteria) (0.2–24%) (Figure 2B). The shifts in the prokaryotic community at class-level were not found to be related to meteorological season or year (Supplementary Figure 2B). Despite the relative consistency in the distribution of bacterial ASVs at class level throughout the sampling period, there was a remarkable difference between 16S libraries at ASV level according to Sorensen similarity coefficient values ranging from 0.06 to 0.41 (Figures 2B, 3). Out of the total 5590 identified ASVs only seven were present in all 16 samples, together representing between 2 and 23% of the relative sequence abundance per sample (Supplementary Figure 5). These were primarily represented by Alphaproteobacteria (Rhizobiales, Rhodobacterales and Sphingomonadales (100% identity with *Porphyrobacter sanguineus*)) and Planctomycetes (Pirellulales), with lower relative abundances of Gammaproteobacteria (Betaproteobacteriales) and Deltaproteobacteria (PB19) (Supplementary Figure 5). On average, 28% ± 6 (20–37%) of the ASVs were detected in a single sample. Similarly, there was no consistency in which ASVs dominated the sequence libraries.

### Correlations of ASV Modules With Temperature and Biomass

**FIGURE 4:**
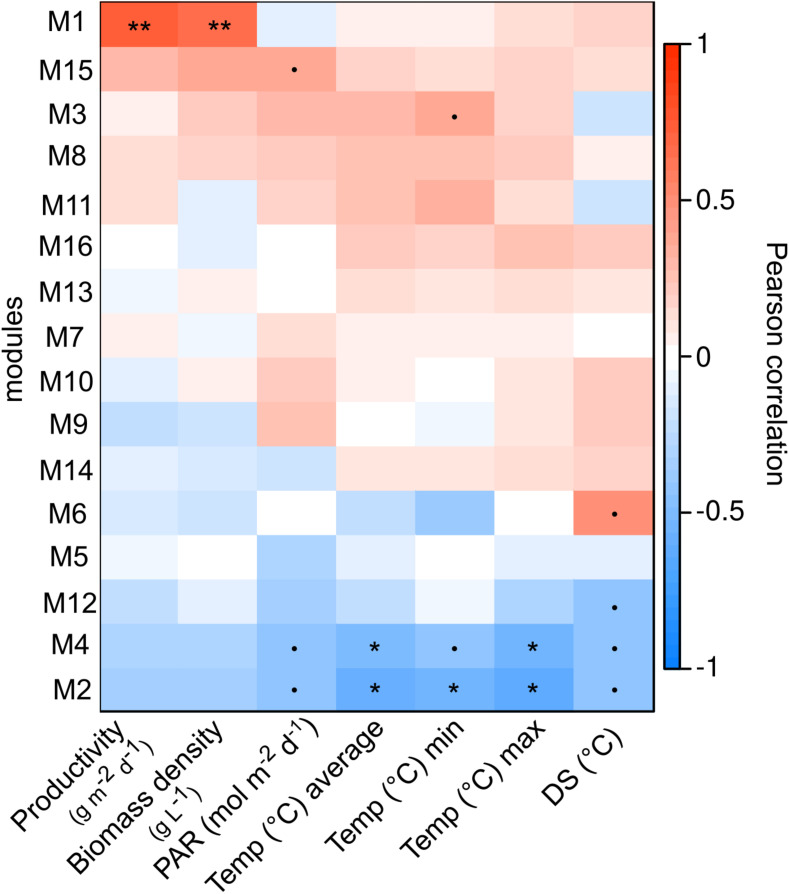
Pearson coefficient correlations calculated between network modules eigenvalues (each module id is indicated to the left with corresponding number) and environmental parameters including productivity (g^−1^ m^−2^ day^−1^), biomass density (g L^−1^), light (PAR, mol^−1^ m^−2^ d^−1^), temperature in panels (°C, average, min, and max) and diurnal shifts in temperature (difference between minimum and maximum daily temperature, DS °C). *P*-values are: *p* < 0.1^•^, *p* < 0.05*, and *p* < 0.01**.

In total, 16 modules (M1-M16) of co-occurent ASVs were found in the network analysis, of which three (modules M1, M2, and M3) had significant (*p* < 0.03) correlations with either temperature or biomass parameters (density and productivity) and ASV relative abundances. The dominant microalgae ASV_18S_1 (*Monoraphidium*) and ASV_18S_2 & 3 (*Mychonastes*) were found in M2 and M3 respectively (Figure 4), with significant (*p* < 0.03) negative correlations (−0.55 to −0.62) with all or one of the temperature parameters (average, min or max) (Figure 4). The relative abundance of ASV_18S_1 peaked at temperature conditions ranging between 7 and 22°C, while the relative abundance of ASV_18S_2 & 3 peaked during both lower (8 & 13°C) and higher (19 & 23°C) mean temperatures. The dates with lower relative abundances had mean temperatures between 13 and 22°C (Figures 1, 4). The bacterial ASVs that were most responsive to temperature along with *Monoraphidium* (ASV_18S_1) were assigned to alphaproteobacterial *Beijerinckiaceae, Devosiaceae, Hyphomonadaceae, Rhizobiaceae* and *Sphingomonadaceae*, together with bacteroidetal *Flavobacteriaceae* and *Spirosomaceae* (Supplementary Table 5). The bacterial ASVs found to correlate with temperature and microalgae *Mychonastes* (ASV_18S_2 and 3) were assigned to *Devosiaceae, Micavibrionaceae, Rhodobacteraceae, Saprospiraceae*, planctomycetal *Pirellulaceae* and *Verrucomicrobiaceae*. Module M1 had a significant positive correlation to both productivity of biomass and biomass density (dry weight) in the PBR (Figure 4) but neither of the dominating microalgal ASVs were found in this module.

## Discussion

Using microbial consortia in microalgal applications is less common than monocultures, partly due to regulations and tradition. Monocultures are kept at a limited range of optimal conditions which can ensure the production of specific compounds such as lipid-rich biomass or certain carotenoids (Cezare-Gomes et al., 2019). However, it has been shown that biodiversity and species richness can increase biomass production and stability of many groups of organisms in the environment globally in both terrestrial, marine and freshwater ecosystems (Cardinale et al., 2011; Duffy et al., 2017). The concept has also been shown for microalgae both in terms of quantity and quality of biomass (Shurin et al., 2014; Jackrel et al., 2018). In this study, we characterized a microbial consortia of microalgae and bacteria, originating from the coastal Baltic Sea, cultivated outdoors for a duration of 3 years in close proximity to a cement factory to capture part of their CO_2_ emissions. We found that the microbial consortia was able to adapt to seasonal and diurnal changes in light and temperature with a sustained capacity to produce biomass at 3.5 g m^–2^ day^–1^ over 41% of the time. This adaptation was manifested by drastic taxonomic shifts among members of different microalgal genera primarily in response to temperature, and a succession of bacterial taxa, which was in part induced by both temperature and diurnal shifts in temperature. The current study is one of the first to show, in a reactor at pilot scale, how a microalgal cultivation system, subjected to large fluctuations in temperature and light over time, can maintain steady biomass production by benefiting from the response diversity of the contained microalgae.

### Temperature as a Main Driver of Biomass Production and Quality

Previous studies have confirmed the influence of environmental parameters on both quality and quantity of biomass in microalgal cultivation systems (Mattsson et al., 2019; Olofsson et al., 2019) but little emphasis has been put on how the presence and abundance of other microbes and their temporal modifications influence such processes. Hong et al. (2017) did however find that species succession influenced both biomass productivity and characteristics of biomass in a raceway mass cultivation system. In our study, the variation in biomass density, biomass productivity as well as high quality product (lipids, proteins and carbohydrates) was found to be closely associated to fluctuations in environmental conditions rather than the succession of microalgal and bacterial taxa. No major impact of microbial succession on either quantity or quality of biomass could therefore be confirmed. The lack of effect of taxonomic shifts on biomass productivity and quality could be a result of functional redundancy (Shade et al., 2012) among both the microalgae and the bacteria, and thus, the same functions may be maintained resulting in the continued production of biomass.

Temperature was the major explanatory factor for the overall performance of the PBR both in terms of biomass density and quality. Variation in temperature is one of the main challenges when it comes to outdoor algal cultivation since it affects the growth rate and biochemical composition (Singh and Singh, 2015; Mattsson et al., 2019) and can therefore challenge the stability. Temperature is an important factor for growth of microalgae and can impact lipid composition, carbon fixation and uptake of nutrients (Juneja et al., 2013). Temperature can also play an important role in photo-inhibition that will ultimately affect the growth (Barati et al., 2019). High temperatures during the day can cause severe temperature stress and decrease the overall performance of the system whereas cold temperature and large seasonal shifts can challenge the ability of the PBR to maintain high and stable productivity (Mata et al., 2010; Khan et al., 2018). González-Camejo et al. (2019) grew indigenous microalgae (dominated by *Chlorella*) in treated effluent from a waste water treatment plant under different seasons with and without temperature control. They found no differences in cultivation performance at temperatures 15–30°C. At temperatures above 30–35°C however, microalgal viability was reduced since the system became ammonium limited as a result of ammonium oxidizing bacteria. Often, temperature is aimed at being kept constant which requires a large energy consumption for the heater/cooler (Pérez-López et al., 2017). Biomass density in the current study differed between the years but was, despite fluctuating temperature, kept at a similar level in the current study compared to Dae-Hyun et al. (2017) that cultivated microbial consortia dominated by green microalgae at a constant temperature of 20°C. The lower biomass density in 2016 compared to the previous 2 years may be a result of the expansion of the system (doubled in volume). A recirculation system for the flue gas was also installed with the aim to reduce the CO_2_ content in the outgoing air, probably resulting in slightly lower carbon supply for the microalgae. The present study shows that even under temperature fluctuations, the biomass productivity was kept at 3.5 ± 3 g m^–2^ d^–1^ throughout the 3 years (Figure 1). To maintain biomass productivity at changing temperature is one of the major challenges when it comes to less controlled systems such as outdoor PBRs. These findings are therefore an important step toward achieving sustainable outdoor cultivation systems, highlighted by Pérez-López et al. (2017), which through life cycle analysis found temperature control to be the main environmental burden in a tubular photobioreactor, located in temperate conditions (Wageningen, The Netherlands).

### Maintained System Function During Shifts in Taxonomy

A community of multiple algal species is thought to increase resilience to environmental fluctuations and hence increase stability of biomass production and quality as a result of functional diversity (Stockenreiter et al., 2013; Nalley et al., 2014; Carruthers et al., 2019). In this study, the mixed microbial consortia alternated between two predominant green microalgal species. These two species were identified as *Monoraphidium* (26–92% of sequences in libraries, ASV_18S_1), that has been observed in 18S sequence libraries from the northern part of the Baltic Sea (Hu et al., 2016), and two ecotypes (99.7% identity, Supplementary Figure 3) of *Mychonastes* (1–55% of sequences in libraries, ASV_18S_2 and ASV_18S_3). Previous studies have found both *Mychonastes* and *Monoraphidium* to be good candidates for lipid production (Yee, 2016) which could increase profit of microalgal cultivation. In the network analysis, *Monoraphidium* (ASV_18S_1) showed a high resilience to temperature since it was found to dominate sequence libraries at temperatures between 7 and 22°C. Most other studies have cultivated *Monoraphidium* at temperatures above 20°C (Yee, 2016). However, a *Monoraphidium* strain (CCALA 1094) isolated from an ice-covered lake, with >99% similarity to ASV_18S_1, grew well at a wide range of temperatures (1–20°C) and irradiances, with the highest growth rates found at 6–20°C (Řezanka et al., 2017). Therefore, *Monoraphidium* could be an excellent candidate for outdoor cultivation in regions with fluctuating temperatures.

ASVs affiliating with *Mychonastes* (ASV_18S_2 and ASV_18S_3) were more abundant at sampling occasions where temperature was high (sampling occasions S1 and S2) or low (sampling occasions S9 and S10). Malinsky-Rushansky (2002) found that a cultured isolate of *Mychonastes homosphaera* had the highest growth rates at 14 and 20°C whereas cell numbers decreased drastically above 28°C. Additionally, a study by Simpson et al. (1978), showed that a strain of *Mychonastes ruminatus* was tolerant to temperatures between 5 and 35°C, with optimal growth at 25–30°C. Thus, by having asynchrony in the response to temperature by different strains in the culture, as in the present study, the stability of production and resilience to face temperature variations could be enhanced. Response asynchrony or crop-rotation has been suggested as a tool to enhance productivity during colder seasons (Butterwick et al., 2005). When using a locally adapted mixed consortia of microalgae that is functionally diverse, similar to the present study, a form of crop-rotation is likely to occur naturally in response to environmental shifts. The observed shifts between the three main ASVs of green microalgae (*Monoraphidium* and *Mychonastes*) could be an example of both the “sampling effect” and the “complementarity effect.”

In addition to its temperature tolerance, some *Mychonastes* strains has an ability for mixotrophic growth. Paranjape et al. (2016), showed that a *Mychonastes* strain, closely related to ASV_18S_2 (KT250608, 99.7% identity; Supplementary Figure 3), increased its biomass production (1.3 fold increase) and growth rate (2.4 fold increase) under mixotrophic growth conditions (in glycerol and constant light), which could e.g. further enhance the possibility for growth at lower light conditions together with the asynchrony in temperature responses. Mixotrophs are able to take up some of the organic carbon derived from their own production and exudation (Kamjunke and Tittel, 2009), and thus could have an advantage over strict phototrophs at carbon limiting conditions. Thus, the compositional dynamics, and consistency in dominating microalgal species over the course of the 3-year study is suggested to be the result of the functional diversity of the two dominating microalgal species within the consortia. Therefore, local microbial consortia with a combination of taxa with complementary functions, could be a good option for an outdoor microalgal cultivation system.

The role of heterotrophic bacteria in algal cultivation systems has been recently explored (Weis et al., 2008; Stockenreiter et al., 2013; Shurin et al., 2014; Beyter et al., 2016; Newby et al., 2016) and suggests that bacteria can be important contributors to enhance stability and productivity. In aquatic systems, bottom-up factors (supply of nutrients) often influence the bacterial succession (Sae-Hee et al., 2020), which could be hypothesized to be true for microalgal cultivation systems as well. With the dominance of a few microalgal species, as in the present study, a selected bacterial community adapted to grow on nutrients from specific microalgal nutrients could be expected to have evolved in the reactor. In our work, there was however no clear indication that the shifts in the microalgal community composition affected the bacterial community composition in the pilot reactor. The fluctuating bacterial community in the PBR also had negligible effect on biomass production. The characterization of the bacterial population showed that the same bacterial classes were dominant throughout the 3 years, belonging to groups commonly found in natural microbial communities of the Baltic Sea, like Alphaproteobacteria, Gammaproteobacteria, Bacteroidia, Planctomycetacia, Verrucomicrobiae and Cyanobacteria (Figure 2B; Andersson et al., 2009; Lindh et al., 2015; Bunse et al., 2016; Hu et al., 2016). Bacteria affiliated to similar bacterial classes as in this study are both a part of the common repertoire of the Baltic Sea and typical in cultivation systems. They have been found in wastewater treatment ponds (Alpha-, Beta- and Gammaproteobacteria, Bacteroidetes (*Chitiniphagaceae, Saprospiraceae*) and Verrucomicrobia) (Wang et al., 2011), and closed bioreactors of varying sizes (5ml to 200L) with a green algal monoculture (*Hyphomonadaceae, Alteromonadaceae, Chitiniphagaceae, Cyclobacteriaceae, Saprospiraceae, Pirellulaceae, and Verrucomicrobia*) (Fulbright et al., 2018), and in biofilms of green algal photobioreactors (*Sphingomonadaceae*, Caulobacterales, *Rhizobiaceae, Rhodobacteraceae, Burkholderiaceae, Flavobacteriaceae, Planctomycetacia* and *Verrucomicrobiaceae*) (Krohn-Molt et al., 2013). The large fluctuations in bacterial ASVs present at different sampling occasions do however suggest that few are tolerant enough to persist over time.

Despite the overall similarity of bacterial classes throughout the 3 years, there were large differences between samples and few overlaps on ASV-level (Figure 3). This suggests that the PBR was a harsh environment for the bacteria and that no bacterial ASV was particularly successful throughout the study period. There could be several reasons for this pattern. Either different bacterial taxa could grow as opportunists but be constrained by the environment in the microalgal cultivation system and therefore replaced by other taxa shortly. In natural Baltic Sea assemblages, opportunistic patterns in bacterial communities have previously been found but the consistent dominance of a few strains can be expected (Lindh et al., 2015). As suggested by Teeling et al. (2012), the availability of algal substrate can provide a series of niches even in seemingly homogenous environments of marine bacterioplankton and therefore induce extinction of certain bacterial taxa by direct competition among the bacteria. A recent study, performed in the same reactor system as this study (Sörenson et al., 2021), suggested that highly productive microalgae, outcompete the bacteria for organic carbon, resulting in increased bacterial diversity. The microalgae from cultivation systems are subjected to high selection pressure as a result of the high production (Shurin et al., 2014), which may release the associated bacteria from a similar pressure, and instead result in a bacterial community with high diversity. Microbial communities with a high diversity in general have a wider functional capacity compared to a community with lower diversity. Thus, the bacterial community in the microalgal cultivation system may have a high functional diversity, which could ensure sporadic opportunistic bacteria to grow. High temperature is another factor that can increase the success of bacteria. González-Camejo et al. (2019) found that when ambient temperatures remained high, the nitrifying bacteria present in the system were able to outcompete the microalgae and collapse the culture. Differential gene expression analyses have also suggested that lower temperatures promote organic carbon uptake by autotrophs (Sörenson et al., 2021). The impact from this highly fluctuating bacterial community on the microalgae in the present study is unclear, however, it does not seem to have a hampering influence on the system.

## Conclusion

The current study shows that a microbial consortium of green microalgae and bacteria, originating from local waters in (cold) temperate zone, provided a stable biomass production over a 3 year-period in an outdoor PBR exposed to constant changes in light and temperature. The performance of the system both in terms of production and quality of biomass was connected to temperature. Shifts between *Monoraphidium* and *Mychonastes* promoted stability to temperature fluctuations. They alternated in dominance with *Monoraphidium* at temperatures between 7 and 22°C and two ecotypes of *Mychonastes* that occurred at low and high temperature respectively. Therefore, as a community, they covered the wide temperature span throughout the cultivation period. The bacteria present in the PBR had a minor impact on the overall performance of the system. We recommend to use local consortia of algae and bacteria that have been adapted to the ambient conditions and therefore are resilient to the environmental changes of light and temperature. This would reduce the need of contamination control and energy intensive heating systems which would contribute to the overall sustainability of outdoor microalgal cultivation.

## Data Availability Statement

The datasets presented in this study can be found in online repositories. The names of the repository/repositories and accession number(s) can be found below: https://www.ebi.ac.uk/ena, PRJEB36394/ERP119583.

## Author Contributions

LM, ES, CL, EL, and HF contributed to conception and design of the study. LM, ES, MH, EL, MO, and FS collected the data. LM, ES, and HF organized the molecular database. LM, EL, MO, and FS organized the metadatabase. MH, FS, and LM did lab analyses. EC supported in network analysis. LM and ES performed the data analysis and wrote the first draft of the manuscript. All authors contributed to methodological matters, manuscript revision, read, and approved the submitted version.

## Conflict of Interest

The authors declare that the research was conducted in the absence of any commercial or financial relationships that could be construed as a potential conflict of interest.

## Footnotes

1 http://strang.smhi.se/
