# Supplementary material for: Functional Diversity Facilitates Stability Under Environmental Changes in an Outdoor Microalgal Cultivation System
Source: Front Bioeng Biotechnol. 2021 Apr 22;9:651895. doi: 10.3389/fbioe.2021.651895 (PMC8100445; doi:10.3389/fbioe.2021.651895)
Supplement: Supplementary file 1 [file Data_Sheet_1.docx]

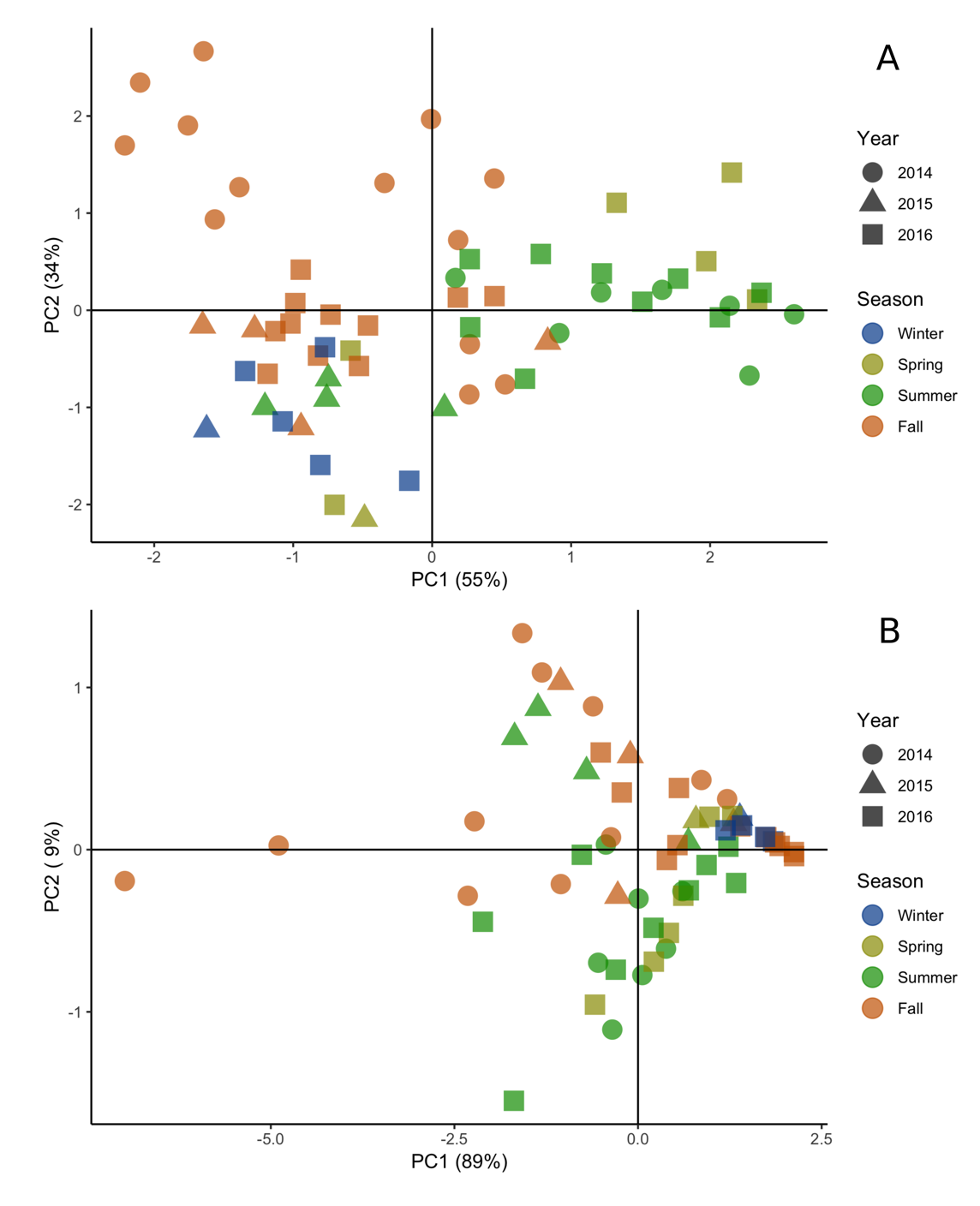


**Supplementary Figure 1.** PCA of high quality products (lipids, protein and carbohydrate) % of dry weight (A) and productivity (g m^-2^ d^-1^, B) for winter, spring, summer and fall during all three years (2014-2016).


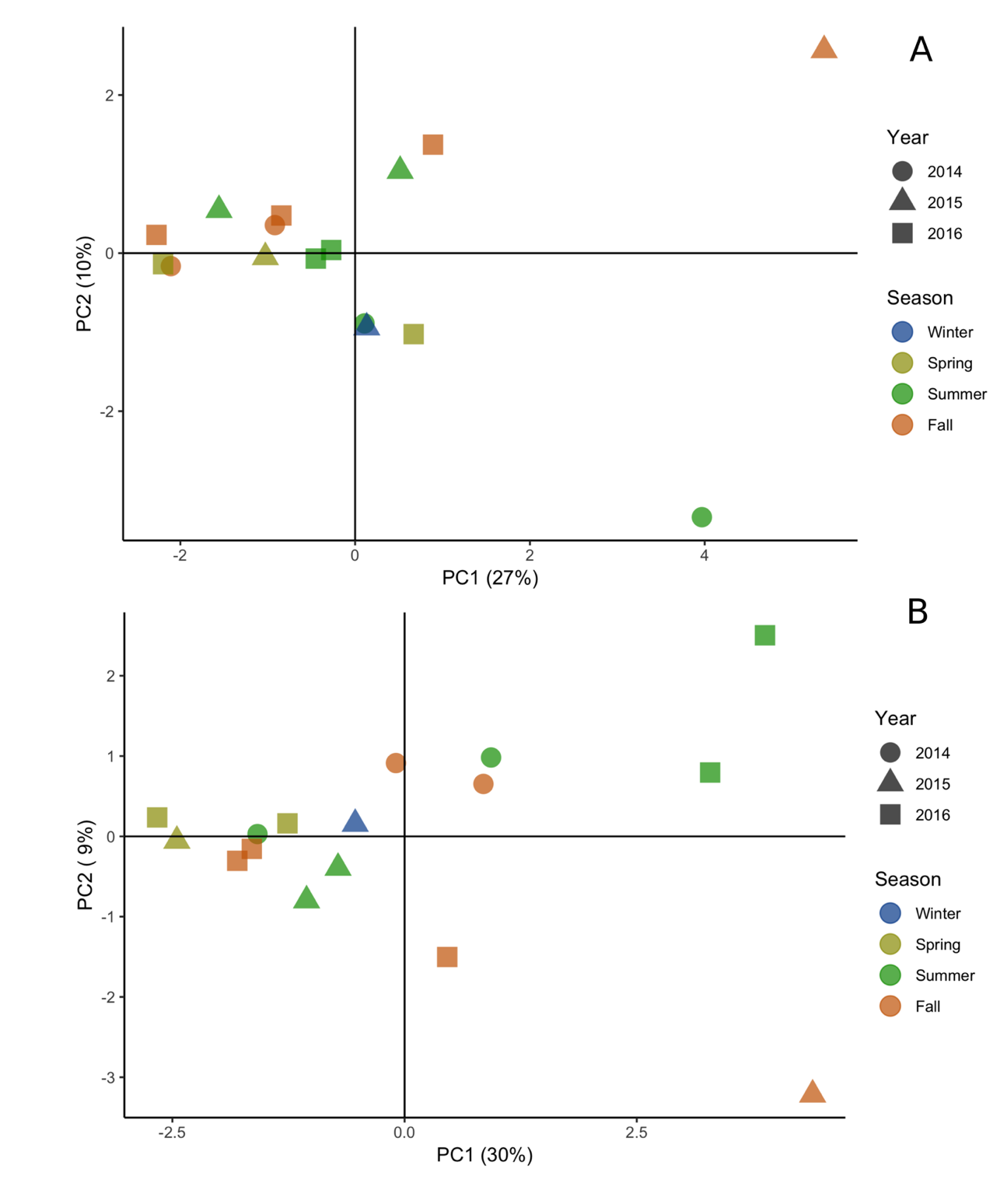


**Supplementary Figure 2.** PCA of 18S (A) and 16S (B) rRNA gene based community using CLR transformation for winter, spring, summer and fall during all three years (2014-2016).


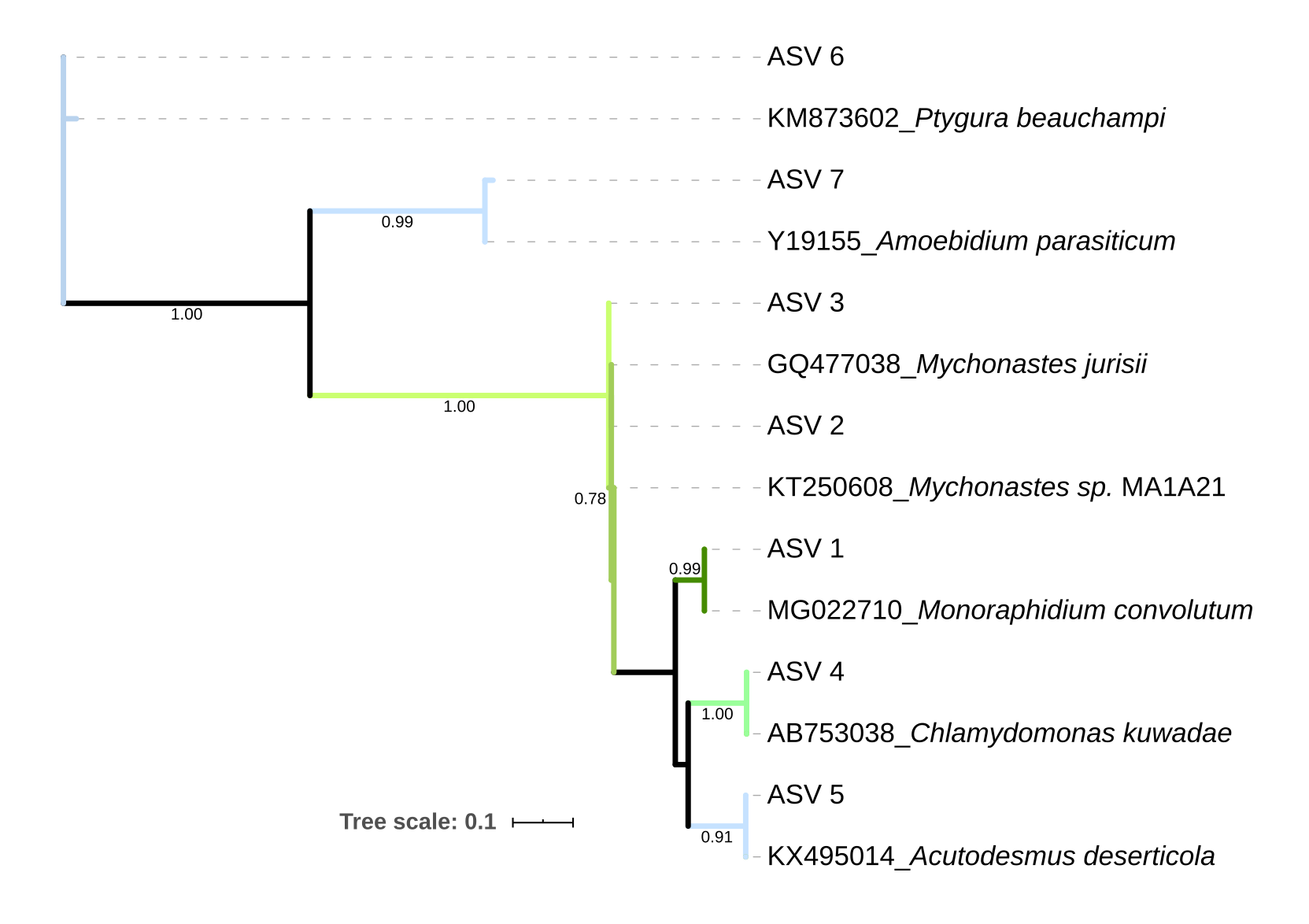


**Supplementary Figure 3.** Maxiumum likelihood tree of top7 18S ASVs, with close hits (GenBank nBLAST), branch support shown >0.75 (bootstraps = 500). Node colours refer to colours given to ASVs in Figure 2.


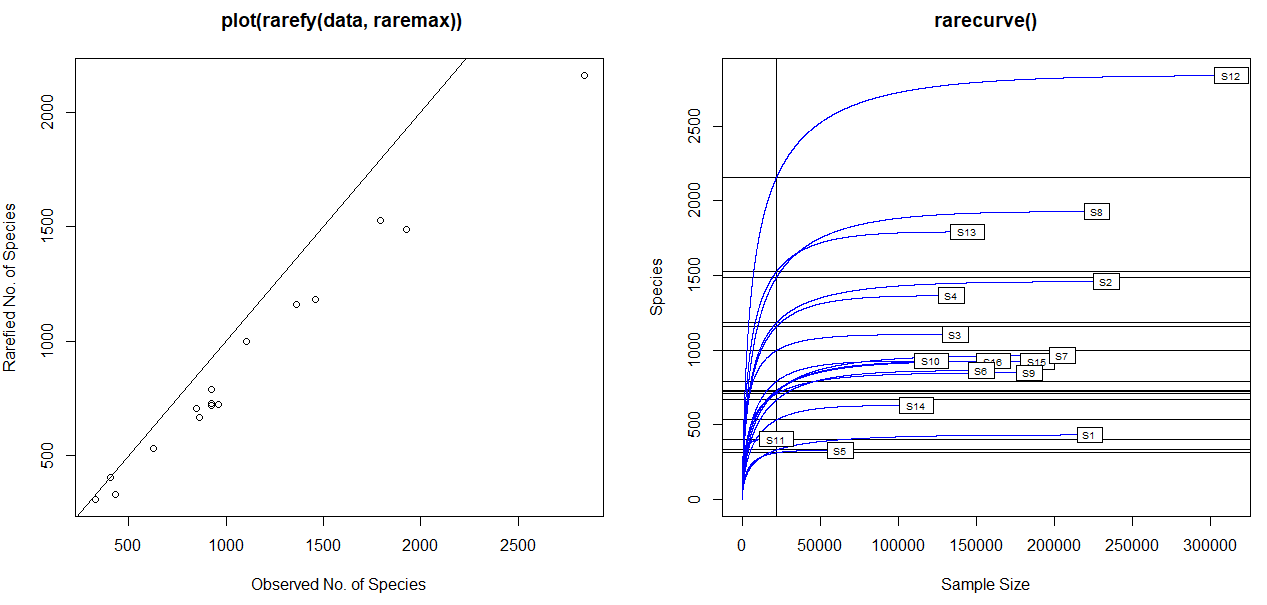


**Supplementary Figure 4.** Rarefaction curve of 16S amplicon data based on ASV-level.


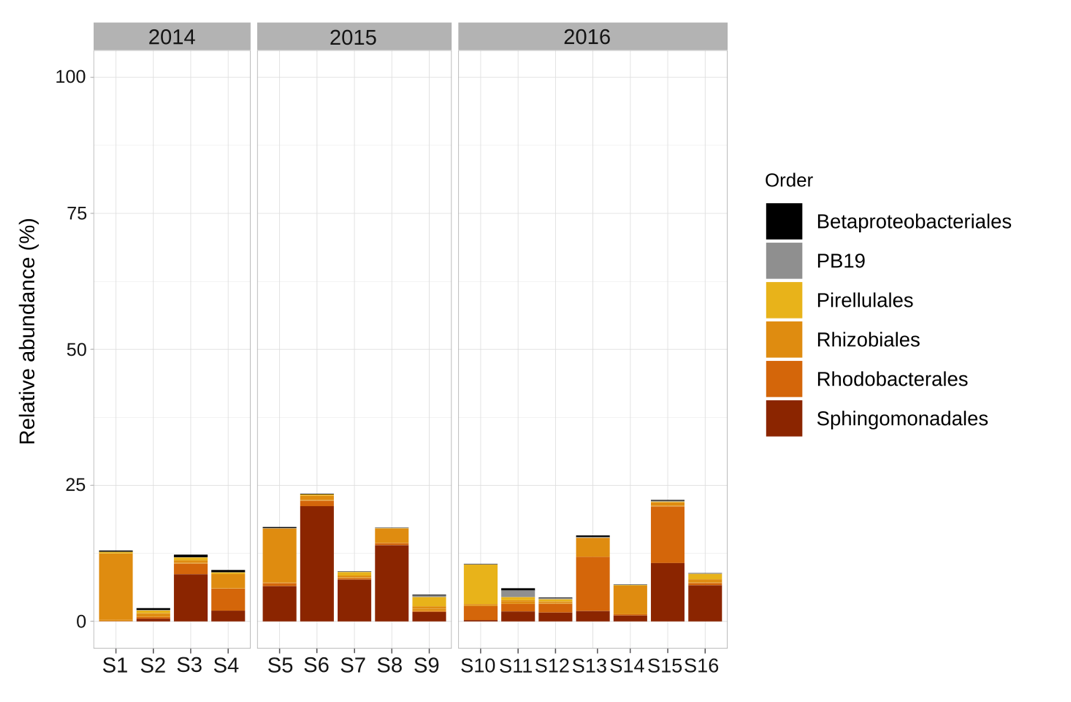


**Supplementary Figure 5.** Core 16S ASVs, present in all 16 samples (S1-S16), at order level.

**Supplementary Table 1.** Biomass metabolite averages ± standard deviation / ranges for the three years (2014-2016). NA’s not included in calculations.

| **Year** | **Lipids** | | **Proteins** | | **Carbohydrates** | |
| --- | --- | --- | --- | --- | --- | --- |
|  | **%** | **g m^-2^ d^-1^** | **%** | **g m^-2^ d^-1^** | **%** | **g m^-2^ d^-1^** |
| *2014* | 32 ± 5 / 24-41 | 2.0 ± 1.4 / 0.5-5.6 | 23 ± 9 / 12-40 | 1.4 ± 1.1 / 0.4-4.5 | 19 ± 10 / 9-37 | 1.3 ± 0.9 / 0.1-4.0 |
| *2015* | 25 ± 3 / 19-29 | 1.0 ± 0.6 / 0.2-1.9 | 35 ± 5 / 26-44 | 1.4 ± 0.8 / 0.4-2.5 | 19 ± 5 / 14-30 | 0.7 ± 0.4 / 0.2-1.3 |
| *2016* | 28 ± 3 / 19-35 | 0.7 ± 0.7 / -0.2-2.5 | 24 ± 8 / 11-40 | 0.5 ± 0.5 / -0.2-1.8 | 20 ± 8 / 10-37 | 0.6 ± 0.6 / -0.1-2.4 |
| *2014-2016* | 29 ± 5 / 19-41 | 1.2 ± 1.1 / -0.17-5.6 | 25 ± 9 / 11-44 | 1.0 ± 0.9 / -0.18-4.5 | 20 ± 8 / 9-37 | 0.8 ± 0.8 / -0.1-4.0 |

**Supplementary Table 2.** Different years and seasons environmental conditions. Averages ± standard deviation / ranges for the three years (2014-2016). NA’s not included in calculations. All data included and therefore also temperature and DS values calculated by conversion factor included.

|  | **Temperature** | **Diurnal shifts in temperature** | **PAR** |
| --- | --- | --- | --- |
| *2014* | 16.0 ± 5.9 / -3.5-29.0 | 6.7 ± 3.5 / 0.05-21.7 | 20.4 ± 18.2 / 0.7-59 |
| *2015* | 13.6 ± 5.4 / 2.1-26.2 | 6.0 ± 3.3 / 0.4-21.1 | 20.5 ± 17.1 / 0.4-58.6 |
| *2016* | 13.0 ± 6.7 / -3.8-24.7 | 6.9 ± 4.4 / 0.08-24.3 | 20.6 ± 17.2 / 0.7-58.8 |
| *2014-2016* | 13.9 ± 6.2 / -3.8-29.0 | 6.5 ± 3.8 / 0.05-24.3 | 20.5 ± 17.4 / 0.4-59 |
| *Winter* | 7.1 ± 3.0 / -3.8-14.9 | 3.5 ± 1.5 / 0.05-6.9 | 4.3 ± 4.4 / 0.4-23.7 |
| *Spring* | 13.5 ± 3.3 / 5.5-20.9 | 8.2 ± 3.8 / 2.3-17.4 | 31.2 ± 13.5 / 3.6-56.7 |
| *Summer* | 20.9 ± 2.2 / 15.8-29.0 | 8.8 ± 3.2 / 1.0-24.3 | 40.0 ± 11.8 / 12.3-59 |
| *Fall* | 15.7 ± 4.5 / 3.7-23.8 | 6.9 ± 4.1 / 0.3-21.7 | 16.1 ± 11.7 / 0.8-42.9 |

**Supplementary Table 3.** Number of sequences in quality control steps of dada2 for 16S sequences in raw counts (including chloroplast and mitochondrial sequences).

| **Sample** | **input** | **filtered** | **denoised** | **merged** | **non-chimeric** | **chloroplasts** |
| --- | --- | --- | --- | --- | --- | --- |
| S1 | 1028392 | 707172 | 707172 | 702129 | 457321 | 234510 |
| S2 | 1041551 | 674360 | 674360 | 664478 | 470637 | 237081 |
| S3 | 428978 | 291837 | 291837 | 286200 | 186572 | 49877 |
| S4 | 781030 | 542694 | 542694 | 535948 | 390493 | 256367 |
| S5 | 489376 | 334317 | 334317 | 331650 | 255981 | 192767 |
| S6 | 793738 | 544892 | 544892 | 537644 | 378180 | 224725 |
| S7 | 1054415 | 720358 | 720358 | 713023 | 507567 | 301888 |
| S8 | 873597 | 607104 | 607104 | 596392 | 396422 | 168992 |
| S9 | 1065694 | 751301 | 751301 | 740788 | 542911 | 358638 |
| S10 | 1099643 | 647385 | 647385 | 641840 | 519500 | 398167 |
| S11 | 56309 | 39640 | 39640 | 37929 | 26958 | 4840 |
| S12 | 997115 | 701076 | 701076 | 680597 | 461160 | 147767 |
| S13 | 681386 | 448289 | 448289 | 440150 | 346436 | 202379 |
| S14 | 724701 | 486335 | 486335 | 482553 | 368487 | 256865 |
| S15 | 1055994 | 710971 | 710971 | 702400 | 518396 | 329161 |
| S16 | 738553 | 493679 | 493679 | 487971 | 343359 | 182070 |

**Supplementary Table 4.** Number of sequences in quality control steps of dada2 for 18S sequences in raw counts. Only forward sequences were used.

| **Sample** | **input** | **filtered** | **denoised** | **non-chimeric** |
| --- | --- | --- | --- | --- |
| S17 | 491480 | 436333 | 436333 | 426087 |
| S18 | 581707 | 518075 | 518075 | 506949 |
| S19 | 133834 | 121268 | 121268 | 120845 |
| S20 | 324803 | 297765 | 297765 | 295007 |
| S21 | 280327 | 255172 | 255172 | 252411 |
| S22 | 324686 | 302006 | 302006 | 300451 |
| S23 | 387732 | 359729 | 359729 | 357927 |
| S24 | 371929 | 339905 | 339905 | 337461 |
| S25 | 436807 | 392105 | 392105 | 383000 |
| S26 | 456941 | 402842 | 402842 | 394319 |
| S27 | 116415 | 100753 | 100753 | 99954 |
| S28 | 304869 | 280752 | 280752 | 278819 |
| S29 | 233093 | 214999 | 214999 | 214013 |
| S30 | 274836 | 254009 | 254009 | 252266 |
| S31 | 398609 | 369979 | 369979 | 368625 |
| S32 | 324362 | 299825 | 299825 | 298626 |

**Supplementary Table 5.** ASVs and their taxonomy of network modules

| ASVs | OTUs | TaxLevel1 | TaxLevel2 | Module  labels |
| --- | --- | --- | --- | --- |
|  | 04f0b10f56a5b853742ea8f18c83ad23 | Bacteroidia | Spirosomaceae | 1 |
|  | adfaa27b6ec366cd3fb9a8f59c3b1582 | Alphaproteobacteria | Rhizobiaceae | 1 |
|  | d6986664b737b35d331ee47afc46f3f4 | Bacteroidia | Saprospiraceae | 1 |
|  | e185c425fbb282aea2e0557ac1f3a0fd | Verrucomicrobiae | DEV007 | 1 |
|  | 55606ab6b5ba2a2130a24cdb77eb34e1 | Bacteroidia | Saprospiraceae | 1 |
|  | e89cc56fb965a6bceeac1fa3da10ec54 | Bacteroidia | Saprospiraceae | 1 |
|  | 8532eac11c34b58c873f9bfe04ec800c | Bacteroidia | Saprospiraceae | 1 |
|  | 9627750df3b2f8a4bdbb0d2b9eea8577 | Bacteroidia | Saprospiraceae | 1 |
|  | 6b361805548c7d1d30711b7f8716401e | Alphaproteobacteria | Rhodobacteraceae | 1 |
|  | 3c3bc2b6faa2b74be539ea310223874d | Gammaproteobacteria | Rhodocyclaceae | 1 |
|  | dfd56c93eddc0a441ffde7068acad4af | Gammaproteobacteria | Halieaceae | 1 |
|  | 25b3650429cdfc11eb8ddc65151dce5c | Gammaproteobacteria | Burkholderiaceae | 1 |
|  | 12752ff812dc6e894dfc8363132d97e1 | Planctomycetacia | Pirellulaceae | 1 |
|  | 0c096d1c5c2916b96eb8761eed02781d | Gammaproteobacteria | Methylophilaceae | 1 |
|  | 63944ed8647b3d8a1267606cf886a176 | OM190 | uncultured_bacterium | 1 |
|  | 92e7fca44d89c1def3af0f4b4d9b90e2 | Bacteroidia | Flavobacteriaceae | 1 |
|  | 96c6b96a3e6f1f66a8c42d9b79e52c0d | OM190 | uncultured_bacterium | 1 |
|  | e97ebd5664f2abf5a27f4392af32691a | Bacteroidia | Saprospiraceae | 1 |
|  | c25233e297a50af6ac53a268982dd1e5 | Verrucomicrobiae | Verrucomicrobiaceae | 1 |
|  | 10b6ebc9c8c2da5526b7c13b4dd14a98 | Bacteroidia | Crocinitomicaceae | 1 |
|  | 4dbf77c9ef1d349b2275dfc2472b3711 | Planctomycetacia | Pirellulaceae | 1 |
|  | 38834194d690f31a6940aedb530f044d | Gammaproteobacteria | NA | 1 |
|  | 0fe8e1b0a13bd78dd774c24fed5de302 | Gammaproteobacteria | Xanthomonadaceae | 1 |
|  | 48988a1ce0e76ccb824c7a686457653b | Bacteroidia | Flavobacteriaceae | 1 |
|  | f9ecf7d7ba9db047734f8243251c12d9 | Bacteroidia | Cyclobacteriaceae | 1 |
|  | c916518b7fc47cb3844ad082914137f2 | Verrucomicrobiae | Verrucomicrobiaceae | 1 |
|  | 84dc5ab2f1c47db34dda6fd1acaa1535 | Gammaproteobacteria | NA | 1 |
|  | e66151c7314514683ec4e5626aebf5b9 | Bacteroidia | uncultured | 1 |
|  | dc0e76d52712c4ada89405e22245cad1 | Bacteroidia | uncultured | 1 |
|  | 3cc9291b8f80dc313c75feca9e005a5a | Bacteroidia | Flavobacteriaceae | 1 |
|  | ccd87ceac4b0fd5922786622188b9ccf | Alphaproteobacteria | Rhodobacteraceae | 1 |
|  | 2915709f20302112643f876ddf4f717e | Verrucomicrobiae | Verrucomicrobiaceae | 1 |
|  | 05e759d9dc83b345b869a325ed354ced | Gammaproteobacteria | Alteromonadaceae | 1 |
|  | ea3d5f28ea8cc4523407ecaad91fb060 | Bacteroidia | Flavobacteriaceae | 1 |
|  | 5109e6b3058d8f30698da3b9aff816f1 | Gammaproteobacteria | Burkholderiaceae | 1 |
|  | f6fed7a946e194f5dd77179883d59764 | Bacteroidia | Microscillaceae | 1 |
|  | 6c02e83cc61150d90bf72a3561063cbc | Phycisphaerae | Phycisphaeraceae | 1 |
|  | 93172ac7bde2aff03e9f1f4f81bc9d8b | Alphaproteobacteria | Sphingomonadaceae | 1 |
|  | 75cbd7cdb833133de64806369515b8d3 | Bacteroidia | Saprospiraceae | 1 |
|  | 541b2ced6a5856098c7c25c4b4d49cfc | Verrucomicrobiae | Rubritaleaceae | 1 |
|  | f4d4580cb8058919692aa213a82fb901 | Bacteroidia | Chitinophagaceae | 1 |
|  | c9bc3f37564c26c426b05b069c96d645 | Deltaproteobacteria | NA | 1 |
|  | e4eccebffad430eac1d392674916a929 | Verrucomicrobiae | Rubritaleaceae | 1 |
|  | 6776c09c680d9d836a98c5316bd83daa | Gammaproteobacteria | Burkholderiaceae | 1 |
|  | e638d59b039b264a28f9716887563352 | Alphaproteobacteria | Rhodobacteraceae | 1 |
|  | 3c177b896d3f7fa38b2f30dfe81d8a31 | Gammaproteobacteria | Burkholderiaceae | 1 |
|  | fc57558fbf4243f8a04f4b7f796d41aa | Bacteroidia | Cyclobacteriaceae | 1 |
|  | d557977f056e04ad93d1236f9d954335 | Alphaproteobacteria | Rhodobacteraceae | 1 |
|  | d4e7461693ef3b743ddad89116a483b0 | Verrucomicrobiae | Verrucomicrobiaceae | 1 |
|  | 729d275b1733439cc6e14391bf410366 | Alphaproteobacteria | Sphingomonadaceae | 1 |
|  | b6652e50575bbcf9c04a2044e985f17a | Bacteroidia | Flavobacteriaceae | 1 |
|  | 8834b7587543023e499579b3c2b050d3 | Bacteroidia | Saprospiraceae | 1 |
|  | 09389038283d7bbcedf0a1cb35a3ac9b | Gammaproteobacteria | Halieaceae | 1 |
|  | cba583bc8e8bd7367323b64675de7f46 | Alphaproteobacteria | Rhodobacteraceae | 1 |
|  | 571af9bf8cdad3d6faab70246888a75d | Gammaproteobacteria | Burkholderiaceae | 1 |
|  | a66b8f7f74bd4626348848eb06db1d00 | Gracilibacteria | uncultured_bacterium | 1 |
|  | 6d55900510b9c82e364789eefd9be407 | Deltaproteobacteria | uncultured_bacterium | 1 |
|  | b112acf1b3a80c17319d9737e68c2fd0 | Bacteroidia | Crocinitomicaceae | 1 |
|  | 983e4c6cf4219cf8834ae8186755f6b9 | Phycisphaerae | Phycisphaeraceae | 1 |
|  | fa00a9d01aab623abfde3804fcdfd3ba | Gammaproteobacteria | Pseudomonadaceae | 1 |
|  | bb4ffb77c52653f4b93ff47427c5363c | Bacteroidia | Cyclobacteriaceae | 1 |
|  | 683991afa11a0639cc73781cf525085c | Planctomycetacia | Rubinisphaeraceae | 1 |
|  | 6eb358cb0ad96c0996dd3a270d21ad37 | Bacteroidia | Cyclobacteriaceae | 1 |
|  | 563dd106fd22a767dfd9417528d404b5 | Bacteroidia | Cyclobacteriaceae | 1 |
|  | 355baecee28b4861bbb0975446ab3951 | Bacteroidia | Flavobacteriaceae | 1 |
|  | 4cdcbced540f507457bea9f0ce96aacc | Bacteroidia | Saprospiraceae | 1 |
|  | e52d1718c5198c33e978d9bc1aed6569 | Alphaproteobacteria | Rhodobacteraceae | 1 |
|  | acbf02ea297742e23d038328aa70a9b2 | Gammaproteobacteria | Alteromonadaceae | 1 |
|  | 81a51ae0eac2015856a2184c44613b13 | Bacteroidia | Saprospiraceae | 1 |
|  | 8112ac2e740378a5f625073b4f7d73b8 | Bacteroidia | Flavobacteriaceae | 1 |
|  | 320686539ea9cb51cf7b1d72b3371e6d | Bacteroidia | Saprospiraceae | 1 |
|  | f55199d3938da4141ef44cd2b40a2767 | Gammaproteobacteria | Burkholderiaceae | 1 |
|  | dddc8111a9fa86bbc59f9dec1e358cb2 | Gammaproteobacteria | Burkholderiaceae | 1 |
|  | 741ec4b30ebc9e22b0445830d906558f | Bacteroidia | Cryomorphaceae | 1 |
|  | e47e174afdf2da09a4649fd1008afcc5 | Gammaproteobacteria | Rhodanobacteraceae | 1 |
|  | 4a50a14f48c6cec990c305f4d639bd4e | Bacteroidia | Flavobacteriaceae | 1 |
|  | 94e92cab78a871f576c54dca4dce745f | Gammaproteobacteria | Burkholderiaceae | 1 |
|  | 0082d9cd372d844e9a4903f7f315dfb3 | Chlamydiae | Parachlamydiaceae | 1 |
|  | ac5a6be4715dcbe1344c283832aad899 | Alphaproteobacteria | Rhodobacteraceae | 1 |
|  | 62b5247cfde4f19b3f10daadb093672c | Bacteroidia | Saprospiraceae | 1 |
|  | 700c42b491a1502c21c51ab6e71cb410 | Bacteroidia | Saprospiraceae | 1 |
|  | 227bede407025a8610ea3e48225c0036 | Gammaproteobacteria | Halieaceae | 1 |
|  | 17c141d32c04f4f509de0a250bfe9e2c | Bacteroidia | Flavobacteriaceae | 1 |
|  | 81af3e13fdf6d813daa4b1d4c0fe9904 | Gammaproteobacteria | Burkholderiaceae | 1 |
|  | 39de1d89590d19a470384aa5817f74b3 | Bacteroidia | Flavobacteriaceae | 1 |
|  | b36434a1b74c01858690871dfddcc42f | Alphaproteobacteria | Sphingomonadaceae | 1 |
|  | 19465ddd82f4b1b9f2b4f1240d55878f | Alphaproteobacteria | Sphingomonadaceae | 1 |
|  | 166088f6034b86af46298ffe4dba14ca | Planctomycetacia | Rubinisphaeraceae | 1 |
|  | c6b27799ff618d124dae3ed0647bb725 | Bacteroidia | Flavobacteriaceae | 1 |
|  | 501874087e68991717a9c693db2884fe | Bacteroidia | Cryomorphaceae | 1 |
|  | ac9afa4d7bc8846d6074686a80f361c4 | Phycisphaerae | Phycisphaeraceae | 1 |
|  | cb57f3df86a25a91aae8fc0a73268ebf | Bacteroidia | Saprospiraceae | 1 |
|  | dba7b319bf128c00ff45bff0bb37e312 | Bacteroidia | Saprospiraceae | 1 |
|  | 8dfea8f27a842e5864a6ed47560f3ab5 | Gammaproteobacteria | Pseudomonadaceae | 1 |
|  | 5fe6fdfb07b17c2710e1a1beee426d9d | Gracilibacteria | uncultured_bacterium | 1 |
|  | 473707092c5648870859322826020e42 | Alphaproteobacteria | Rhodobacteraceae | 1 |
|  | 5f9b70976373268c5858daf0f2473638 | Bacteroidia | Saprospiraceae | 1 |
|  | 40abf4676ddfa4a74fcc67158aeb7407 | Bacteroidia | Flavobacteriaceae | 1 |
|  | 348005014ef2d646dfb2fe42d9df9141 | Alphaproteobacteria | Rhodobacteraceae | 1 |
|  | d7885d7424110bbb221f5ee9ec7a3e8c | Alphaproteobacteria | Rhodobacteraceae | 1 |
|  | 1e561bf27a6e4b91cb995213feb44a8b | Bacteroidia | Saprospiraceae | 1 |
|  | a78c34454d80fd7808213861ef353fb3 | Alphaproteobacteria | Hyphomonadaceae | 1 |
|  | eec1c6fd9b50832b37241b25b0f7003a | Gammaproteobacteria | Burkholderiaceae | 1 |
|  | 416c2ff1886de068edd0605026582f46 | Planctomycetacia | Pirellulaceae | 1 |
|  | 5a46e8cf33611ecb85a6691079fcbee3 | Gammaproteobacteria | Burkholderiaceae | 1 |
|  | 3a104b1a56b4d526e03411611a7e90a9 | Bacteroidia | Flavobacteriaceae | 1 |
|  | 4a2a73ce7fe46dd949dcb4a680412ed8 | Gemmatimonadetes | Gemmatimonadaceae | 1 |
|  | 6408e09ef36e4168ec9140e57dfd9506 | Bacteroidia | NA | 1 |
|  | a86040368749441d02e7b67df9b6ba84 | Alphaproteobacteria | Rhodobacteraceae | 1 |
|  | d558dde321138a2cb7dc8ee66913cf79 | Bacteroidia | Flavobacteriaceae | 1 |
|  | 11044737ab55d71da4590a5f8650bcdf | Bacteroidia | Cyclobacteriaceae | 1 |
|  | 424cafa60347c7a82a895295865114df | Chloroplastida | Chlorophyta | 1 |
|  | 8eb3b4d3feaa96a82fd52fd7847e7293 | Alveolata | Ciliophora | 1 |
|  | da06911c57ed12e64b21c7b824d383d1 | Chloroplastida | Chlorophyta | 1 |
|  | 30c07fb973a105c87c4109dc756ada25 | Chloroplastida | Chlorophyta | 1 |
|  | a7199601a56a8bf3bd1c44769e52cf93 | Chloroplastida | Chlorophyta | 1 |
|  | da692b9d9e4c49afa058b864295d12c7 | Alveolata | Ciliophora | 1 |
|  | 6e0e64e31016a56798b70b812548df98 | Chloroplastida | Chlorophyta | 1 |
|  | e77131b783ebb145a4f49fbd0e2371f6 | Nucletmycea | Fungi | 1 |
|  | 3d7d7c92bc5eb9ff2eb5458bbc9c7da9 | Discoba | Discicristata | 1 |
|  | 803889c3f365eafd50171a8928f2951f | Alveolata | Ciliophora | 1 |
|  | 08d4808e21344345bbd4ca0a2fb0d5ae | Nucletmycea | Fungi | 1 |
|  | b1bd923ab8049c1eccf38f41ee296d41 | Pavlovophyceae | Pavlova | 1 |
|  | d936cdf5f3dbe07e8503b1660ff7090a | Bacteroidia | Spirosomaceae | 2 |
|  | e0e07ff65a09bbd05975616ac6840a2c | Bacteroidia | Flavobacteriaceae | 2 |
|  | d22561da057661449b667d23fc98ddd7 | Verrucomicrobiae | Puniceicoccaceae | 2 |
|  | 817b89d0c527671e012fbd9c24801ffc | Bacteroidia | NS11-12_marine_group | 2 |
|  | 874d3202f7bd1e29b6c480ddc5504eea | Bacteroidia | Cyclobacteriaceae | 2 |
|  | cef7b7ca6442c595ff247c5f90fbec5d | Alphaproteobacteria | Rhizobiaceae | 2 |
|  | 7e62fcc06aa0e29beb2fd16c61a70aaf | Gracilibacteria | uncultured_bacterium | 2 |
|  | 57aeeb6ca754b49439d4a48d9d024cc1 | Bacteroidia | NA | 2 |
|  | 004e6adc8707f33bb1e0c9cad09f57a3 | Alphaproteobacteria | Rhizobiaceae | 2 |
|  | b58a9dedc595ab8277245426431586e4 | Saccharimonadia | NA | 2 |
|  | 78bbccd881fb6547e2a14e1aa0e2481c | Verrucomicrobiae | Verrucomicrobiaceae | 2 |
|  | 51804e23c198b62278e90be8c106b27a | Gracilibacteria | uncultured_bacterium | 2 |
|  | f202a7d66feb435b9527c285afe9aa85 | Parcubacteria | NA | 2 |
|  | 34788f305e57b69dc10de44fca8ec3ed | Alphaproteobacteria | Hyphomonadaceae | 2 |
|  | 3a3964be708220f337c1ca20f9b54ecc | Bacteroidia | Flavobacteriaceae | 2 |
|  | 7d1fcac0ce2c463c9b22b2cee928bbbc | Deltaproteobacteria | Bacteriovoracaceae | 2 |
|  | 93153680724601103fc3ddff42c96671 | Alphaproteobacteria | Rhizobiaceae | 2 |
|  | 15e73cf518b466be4053252930d10613 | Verrucomicrobiae | Verrucomicrobiaceae | 2 |
|  | 25c0922dce58fcd82759f16f3a2c0338 | Alphaproteobacteria | Devosiaceae | 2 |
|  | d4683ddea6401dda3618b61314b8f1b5 | Alphaproteobacteria | Sphingomonadaceae | 2 |
|  | 672b4bddafabeaa16d9de16c45d474c7 | Alphaproteobacteria | Rhizobiaceae | 2 |
|  | fee1efd4cece57b470dc7ebb70878c45 | Alphaproteobacteria | Sphingomonadaceae | 2 |
|  | 2dc25f36a64d780d49a71d44db8965a8 | Alphaproteobacteria | Rhizobiaceae | 2 |
|  | b49e5aa37cdc82176d9ec71445bbddb5 | Bacteroidia | Flavobacteriaceae | 2 |
|  | 57c68cebf73d7aab104beb672a11f97b | Bacteroidia | NA | 2 |
|  | 890b7f4b7334510ea127e8b59f859f92 | Alphaproteobacteria | Rhizobiaceae | 2 |
|  | 29f0138e87b7a0212464e83b65586e33 | Alphaproteobacteria | Hyphomonadaceae | 2 |
|  | c53f65c289bc30a6cf498b85e78d5751 | Bacteroidia | NA | 2 |
|  | c739ae8b10662ca5e6d89ec67b718d83 | Bacteroidia | Flavobacteriaceae | 2 |
|  | 3e21f4a51d9ef6d8da685d90f993fa92 | Bacteroidia | Saprospiraceae | 2 |
|  | 9b084199ae3bc839a640f5f77905f875 | Bacteroidia | Cyclobacteriaceae | 2 |
|  | b430e99ded9c0c82413055acbecfa3fc | Alphaproteobacteria | Rhizobiaceae | 2 |
|  | 793ec2485e1128fa456ec659efc29771 | Bacteroidia | uncultured | 2 |
|  | 14429e934500ad3e774ef718fc8887ec | Alphaproteobacteria | Sphingomonadaceae | 2 |
|  | 99f1086eceda6f958d56b257c89b0460 | Alphaproteobacteria | Hyphomonadaceae | 2 |
|  | 6b32bfa42d3c97a7e43af3a1d851c70a | Bacteroidia | Flavobacteriaceae | 2 |
|  | ed8050a931bbc003ed4a92759fd3427c | Saccharimonadia | NA | 2 |
|  | c75dc92d20c26e930ed4f02013135017 | Alphaproteobacteria | Devosiaceae | 2 |
|  | e5987c555380d30da64073b29164f5c6 | Alphaproteobacteria | Sphingomonadaceae | 2 |
|  | 45591e5dddb5044cbb2c7b08610fb458 | Alphaproteobacteria | Hyphomonadaceae | 2 |
|  | 6379fdc65c8c42b5ef86877d5a5da88d | Parcubacteria | NA | 2 |
|  | b4656a2445d41518fcee0b7783f2589a | Alphaproteobacteria | Hyphomonadaceae | 2 |
|  | 19cbc03578dc0176023dbcbb5c6ff9ee | Parcubacteria | NA | 2 |
|  | 32dc693fd52e3b0034bf6b0e1844d829 | Alphaproteobacteria | Rhizobiaceae | 2 |
|  | bdb0024c6d94ab50223a7d4acc36c9d4 | Bacteroidia | NA | 2 |
|  | 3114219a1b3776b240289a2adaa4b225 | Alphaproteobacteria | Beijerinckiaceae | 2 |
|  | 4ea57140a2bbb41993820585027a39d9 | Bacteroidia | Spirosomaceae | 2 |
|  | 59c104648e9c4983b1f4277ea55200f5 | Chloroplastida | Chlorophyta | 2 |
| ASV_1 | c95ba3640f65550dba96d6f781f7d987 | Chloroplastida | Chlorophyta | 2 |
|  | 1e664bc627340e4b0b569e430d6c53ac | Chloroplastida | Chlorophyta | 2 |
|  | ef99eaafbaeb32673a64f263695557b1 | Chloroplastida | Chlorophyta | 2 |
|  | b0ce264a4a8b820b1cb5b39249b2d08d | Rhodophyceae | Rhodellophyceae | 2 |
| ASV_6 | 97630e4c66bc4e362697cce49d046aae | Holozoa | Metazoa_(Animalia) | 2 |
|  | b3ea8faf6d99b090eccf7667715f6956 | Chloroplastida | Chlorophyta | 2 |
|  | 1e59cbe447c78dd358ee04105aa2adbc | Stramenopiles | Bicosoecida | 2 |
| ASV_7 | 17367c35640c266429633f4f923dd6a2 | Holozoa | Ichthyosporea | 2 |
|  | 08d845da02821dade3ac7a493de7143e | Alveolata | Ciliophora | 2 |
|  | 7ae64dd059e2db159a0acf907ff0822b | Holozoa | Metazoa_(Animalia) | 2 |
|  | fffa6dc2ed4ac3e8ed5592e79f3b038e | Alveolata | Ciliophora | 2 |
|  | 0573539fe156337ff7dc1f53bec39bd7 | Planctomycetacia | Pirellulaceae | 3 |
|  | 4bfa43ee982c9c25d776e5a9020d9c3b | Verrucomicrobiae | Rubritaleaceae | 3 |
|  | 2d07f57c29f911a3b215f387d279eb47 | Alphaproteobacteria | Sphingomonadaceae | 3 |
|  | 8f767afacac6624cc1c27af2d1369e71 | Bacteroidia | NA | 3 |
|  | 21c04b94ddf9fe769f8368317448d43e | Gracilibacteria | uncultured_bacterium | 3 |
|  | bd052d50cf5285eeab93698096767b7f | Bacteroidia | Saprospiraceae | 3 |
|  | b14d1725a680202ba5338e1c54f7d6a6 | Bacteroidia | Saprospiraceae | 3 |
|  | efa3699e43030f9e09d9c65000403cb6 | Alphaproteobacteria | Rhizobiaceae | 3 |
|  | 607cc4ad2fd49a579607488df60e2049 | Alphaproteobacteria | NA | 3 |
|  | 6c62e65343f0b2b7b94841d38cc384ba | Anaerolineae | Caldilineaceae | 3 |
|  | 490df57e42f0b11bc8e31dcc4f8f1ef9 | Verrucomicrobiae | Rubritaleaceae | 3 |
|  | 25dbeeb256ecc05607c63045a11eef18 | Acidimicrobiia | NA | 3 |
|  | 2ca8d514fdb15ff298c183490c6c523d | Oxyphotobacteria | NA | 3 |
|  | 7acb40aaf80e268042da136e8514f44b | Bacteroidia | NS11-12_marine_group | 3 |
|  | 0ebe257c20574a4f42be3cfb210220e8 | Bacteroidia | Flavobacteriaceae | 3 |
|  | 19c7deedc9df60c704aa16476d64b3c7 | Deltaproteobacteria | Bdellovibrionaceae | 3 |
|  | 25cdebb6f950c7c22104d64681db7e4c | Bacteroidia | Saprospiraceae | 3 |
|  | 258a1e9ddcb108f6e9f3b7e3aade4599 | Bacteroidia | NS11-12_marine_group | 3 |
|  | 73f7a812c869f93cc3cbe54ab1294f5f | Chlamydiae | NA | 3 |
|  | 5743a2cefcb981e750ad94c675ea50b4 | Alphaproteobacteria | Beijerinckiaceae | 3 |
|  | 8277bf4bae2b39a5c67bea39f7173f1b | Bacteroidia | Saprospiraceae | 3 |
|  | 9ab1162a736ca7655c52342c2acb520f | Deltaproteobacteria | Bdellovibrionaceae | 3 |
|  | 37ddb40c7c3dce8c6669e0c1b0df2e36 | Bacteroidia | Saprospiraceae | 3 |
|  | 1f307d207babbe111aa7b8f8bc860730 | Deltaproteobacteria | Oligoflexaceae | 3 |
|  | 4d853f3e4b53fa68b9f1241884f94b7a | Alphaproteobacteria | Rhizobiaceae | 3 |
|  | 39badab6881d753158ffe4dae7c1ef75 | Stramenopiles | Ochrophyta | 3 |
|  | 7022712a5252081838b7f26513664a40 | Stramenopiles | Ochrophyta | 3 |
|  | 997ce9e4f041241cae80c2e43aaf44ff | Stramenopiles | Labyrinthulomycetes | 3 |
|  | fee10c300a135dcdef2aae06ad5b3df5 | Pavlovophyceae | Pavlova | 3 |
|  | 801efa7602c5f7326db73a218d2bfd30 | Stramenopiles | Bicosoecida | 3 |
|  | 0b7e00a27f08c81e8b005197219fead6 | Stramenopiles | Ochrophyta | 3 |
|  | 3d17176b9f38f3a476d30190dcaa0459 | Nucletmycea | Fungi | 3 |
|  | ca539443838fd7b9a2b7795738028dcb | Discosea | Flabellinia | 3 |
|  | 99cce1102d35094da677a1d73627e3d2 | Alveolata | Ciliophora | 3 |
|  | f72d0627cecaa847888f610721661623 | Nucletmycea | Fungi | 3 |
|  | f6f77a0af370a5123f0cf9920e9990b0 | Chloroplastida | Chlorophyta | 3 |
|  | dd7e2fd63fe75ac64627884df0c14c98 | Alveolata | Ciliophora | 3 |
|  | 116ccd69fe24cd31bb0233d952536f97 | Alveolata | Ciliophora | 3 |
|  | a70844b0217695e925588ef4b5233ab5 | Alveolata | Ciliophora | 3 |
|  | 70375ad7a8fdef77b869111226f9ab2d | Discosea | Flabellinia | 3 |
|  | a38737af0e7c744d20899302a616270c | Rhizaria | Cercozoa | 3 |
|  | 51035bd03ef20282b43909f7256ed975 | Alveolata | Ciliophora | 3 |
|  | 78dcddcf8cdc2de57dd0c3f949d31633 | Alveolata | Ciliophora | 3 |
|  | 2465e7bf62b548088a6e9c92c0079501 | Rhizaria | Cercozoa | 3 |
|  | 1b70a2e079abd689972653d00c7b0b4d | Stramenopiles | Ochrophyta | 3 |
|  | dfc3d4d830069dabe229370497aedf02 | Holozoa | Metazoa_(Animalia) | 3 |
|  | 92a9c383329f9e892a093316d0904013 | Discosea | Flabellinia | 3 |
|  | 17becdb7a31478de1fda8ef9babf56cf | Nucletmycea | Fungi | 3 |
|  | 759882e11ccef6b32fc0ec20db1f36d6 | Alveolata | Ciliophora | 3 |
|  | 64820eca82792b3723d5a97fa54d5fe8 | Alveolata | Ciliophora | 3 |
|  | ff51a2b6d12c7860403343b9350ca2cb | Alveolata | Ciliophora | 3 |
|  | 624a9881738956b026fe651e761c830f | Stramenopiles | Ochrophyta | 3 |
|  | 4e011ceebb568a0b26109d0ef3cc4505 | Stramenopiles | Ochrophyta | 3 |
|  | e263375e80942a5c52d6192ccdbfdf56 | Bacteroidia | Saprospiraceae | 4 |
|  | 67095640e748dd02a98f1e4464c4e55d | Planctomycetacia | Pirellulaceae | 4 |
|  | 6090fe8a3acd997879077d4501dce85d | Alphaproteobacteria | Devosiaceae | 4 |
|  | 1bd773338f51e623e65f533f9e8b7333 | Alphaproteobacteria | Rhodobacteraceae | 4 |
|  | f9cf19d9f61f6b64e8037b27dd37b5be | Verrucomicrobiae | Verrucomicrobiaceae | 4 |
|  | b27bffe351049e7d48576b64122a8c4d | Anaerolineae | Caldilineaceae | 4 |
|  | 06627f003ac098eb33bd873be2a4170c | Gammaproteobacteria | NA | 4 |
|  | af6bcf3f597872aca1f930640300b9a9 | Bacteroidia | NA | 4 |
|  | e99150cc5bb64fa1155e3d39affba8df | Parcubacteria | uncultured_bacterium | 4 |
|  | 4c64c511aa5a329ae0027329c39159ca | Alphaproteobacteria | Rhizobiaceae | 4 |
|  | 0a3905495535e4c0d478c89840430339 | Parcubacteria | NA | 4 |
|  | 5a9850662bf8ee6ce7f566871f13e016 | Anaerolineae | Caldilineaceae | 4 |
|  | 6ab9bdfeb803135fce1640cdfd9642b4 | Planctomycetacia | Pirellulaceae | 4 |
|  | d4f665594153f0fad49100272766c6a4 | Alphaproteobacteria | Rhodobacteraceae | 4 |
|  | 4b67759315cb320347e5572c708683e9 | Gammaproteobacteria | Unknown_Family | 4 |
|  | c0e03f9262c85405bf1a074a65bd5de2 | Bacteroidia | Saprospiraceae | 4 |
|  | 72cf2c1f7dd64025255bb1fdcc8edc62 | Saccharimonadia | uncultured_bacterium | 4 |
|  | 6b0e33af26d48242080b67db2fc1f727 | Verrucomicrobiae | Verrucomicrobiaceae | 4 |
|  | d5113521a33abba03fed00dbf8d63b76 | Parcubacteria | uncultured_bacterium | 4 |
|  | dcdcdc5f8ab3d6a43ed1d42f756a6b3b | Gracilibacteria | NA | 4 |
|  | 2b1b952838bc8fe081bee978b2f87e21 | Gammaproteobacteria | Halieaceae | 4 |
|  | a5c33de9ad5a27b695786c74b94388b0 | Parcubacteria | uncultured_bacterium | 4 |
|  | effd7c1501873d9eadc835a76ae4323c | Deltaproteobacteria | Oligoflexaceae | 4 |
|  | aff130d42985810319eeeb21a4aae950 | Alphaproteobacteria | Rhodobacteraceae | 4 |
|  | f0f14f57381b54a002d856fc4ff5c39a | Planctomycetacia | Rubinisphaeraceae | 4 |
|  | 6546c670e65ee1e8027c8cbb4fd6e502 | Alphaproteobacteria | Rhizobiaceae | 4 |
|  | 52f59831fc88ae4cc2b7a56990f46fad | Gracilibacteria | NA | 4 |
|  | 047b4a56baf1e29dc5f4bee6c9e38e4f | Bacteroidia | Sphingobacteriaceae | 4 |
|  | 4e3d50d618184657dc25a4773c652500 | Bacteroidia | NA | 4 |
|  | 0976dea77c2f3dcbf081336f5daf120a | Verrucomicrobiae | Puniceicoccaceae | 4 |
|  | 60b95e77fc79fc11e561f63c2421e019 | Deltaproteobacteria | Oligoflexaceae | 4 |
|  | 63dc137a1c9d867536566f07e8bda304 | Verrucomicrobiae | Verrucomicrobiaceae | 4 |
|  | f981f513a37cbd7ebea1dca3f16d72c5 | Stramenopiles | Ochrophyta | 4 |
| ASV_3 | 09b764f9b4438ee681785ce54494b6af | Chloroplastida | Chlorophyta | 4 |
|  | 9c748ca4e139ffbda6258197ebfa95a0 | Chloroplastida | Chlorophyta | 4 |
| ASV_2 | c1e00734da7078894efa9b245d27afa6 | Chloroplastida | Chlorophyta | 4 |
|  | 70548658a055b8c43e8d5e49ccacc5cb | Chloroplastida | Chlorophyta | 4 |
|  | f88fa6f2c26d36ed3f0cee5cd0f5bdb5 | Chloroplastida | Chlorophyta | 4 |
|  | c97262cb6ba91524177b743cbb391022 | Chloroplastida | Chlorophyta | 4 |
|  | e109db523a168aea3838a655577fa398 | Chloroplastida | Charophyta | 4 |
|  | d0e80450cb19ec5e54515dc8690e1797 | Rhizaria | Cercozoa | 4 |
|  | 53c16367e2115f044a31c3d4498c0483 | Stramenopiles | Bicosoecida | 4 |
|  | ff05c6391085e1a4cd82c040e24fc746 | Alveolata | Protalveolata | 4 |
|  | 406196ef5a38776fedf7eef1fbd9c26a | Holozoa | Metazoa_(Animalia) | 4 |
|  | d1d28249645ae77432cd3eecc7188779 | Discoba | Discicristata | 4 |
|  | 6d974b4066331202f609a1edfa77ae20 | Holozoa | Choanoflagellida | 4 |
|  | 1ee75b7e14e9fd921099b9522a2182a4 | Holozoa | Choanoflagellida | 4 |
|  | 93bce58bbd52b58ef41ec85ab5450e94 | Holozoa | Choanoflagellida | 4 |
|  | 53894d9316039fba6427fa1b2d862b29 | Alveolata | Ciliophora | 4 |
|  | 983e2e93237554b3f2571fae8119f3ce | metagenome | metagenome | 4 |
|  | 7d456d7a491559dd0a3583caf2dcc3e2 | OM190 | uncultured_bacterium | 5 |
|  | a19866c9379e4969cb9480e6e27d2baf | Alphaproteobacteria | Rhizobiaceae | 5 |
|  | 1b700f5cf69f2db23064b95c8545f33d | Alphaproteobacteria | Sphingomonadaceae | 5 |
|  | c00f12e32685d401394ef9d9adf49c82 | Bacteroidia | Crocinitomicaceae | 5 |
|  | e69080ce20a47b312cec965962545e3b | Alphaproteobacteria | Rhodobacteraceae | 5 |
|  | 8e986372ca0e425e64962dfbf949ff33 | Alphaproteobacteria | Sphingomonadaceae | 5 |
|  | b5392bbea063d9b024a18f99ff968ba4 | Planctomycetacia | Pirellulaceae | 5 |
|  | e46b667081216d357f035cab49d85bcd | Alphaproteobacteria | Rhizobiaceae | 5 |
|  | cda2197b95d4fe04e0d7f99998ddbf29 | Deltaproteobacteria | Bdellovibrionaceae | 5 |
|  | e70a1e7065e031ef54009cb1cf931adf | Bacteroidia | Chitinophagaceae | 5 |
|  | 86c214e542671a7de50c5d8f99fa9d0c | Oxyphotobacteria | Nostocaceae | 5 |
|  | a6afdc9e8e717cb3e7dccf60918f0ca1 | Planctomycetacia | Pirellulaceae | 5 |
|  | 10750f2b873cbfc13a0fb05d12f07a61 | Bacteroidia | Saprospiraceae | 5 |
|  | de4fb533fc88afd48a4e7a828f34a756 | Alphaproteobacteria | NA | 5 |
|  | 76116468db89b1b9d78d64cfcddef1b3 | Bacteroidia | Saprospiraceae | 5 |
|  | c53314a8b784cf9207a0c8f47ea567b6 | Bacteroidia | Flavobacteriaceae | 5 |
|  | 3d4b1f71a07df46850d13910feb2aedf | Alphaproteobacteria | Hyphomonadaceae | 5 |
|  | fbc217822842d7b460a70c2f8f7c853f | Planctomycetacia | Pirellulaceae | 5 |
|  | 1ef61d63d342f8d00ade362439f22e76 | Oxyphotobacteria | Nostocaceae | 5 |
|  | 5a05699e6aec7175654676072348de99 | Bacteroidia | NS11-12_marine_group | 5 |
|  | 57df2b235c2e9aae02c3ab6327e3ce5e | Bacteroidia | Flavobacteriaceae | 5 |
|  | 99cb6326e3c6f676e14183db70f6730f | Stramenopiles | Ochrophyta | 5 |
|  | 7b61a958ce1c0ae1e410743c59b48ca9 | Chloroplastida | Chlorophyta | 5 |
|  | 00b3786f2b5d6d9daf5847d496590a82 | Chloroplastida | Charophyta | 5 |
|  | 2c0097925cabdb57b8c830376dfe6363 | Alveolata | Ciliophora | 5 |
|  | cce2a8ddbbf80f26d405df97d40ee763 | Stramenopiles | MAST-12 | 5 |
|  | 15c5a0b183bd3a57cdc4894bc1995ffa | Chloroplastida | Chlorophyta | 5 |
|  | 22403a552a7e8a178478ff51d06ccd6c | Nucletmycea | Fungi | 5 |
|  | 9f20fb78f7081d3ea05bd04dd21b0f5f | Holozoa | Metazoa_(Animalia) | 5 |
|  | 0a0bf0cef509b99c3b845f221c0e97c9 | Chloroplastida | Charophyta | 5 |
|  | c8be1e641df85ac2619b927305d53a8a | Alveolata | Ciliophora | 5 |
|  | cc5ce88f20dea9807bfdee9bb814e5fb | Rhizaria | Cercozoa | 5 |
|  | f2baf7892cf20a98cf648a750e232525 | Rhizaria | Cercozoa | 5 |
|  | 953ed294b8cd80136060afb5eab1876e | Rhizaria | Cercozoa | 5 |
|  | b2a9aa9afa1aeaf88641b814ed51453d | Nucletmycea | Fungi | 5 |
|  | a0bbe888978f0aba99478d31e107b268 | Alveolata | Ciliophora | 5 |
|  | 62daa5f8b873ce5a6b088e2df68515e5 | Nucletmycea | Fungi | 5 |
|  | 87999a108c832c7480ccee0e9fb9d3f8 | Nucletmycea | Fungi | 5 |
|  | d6f300acfaec834a6907b6bab4fdfca8 | Rhizaria | Cercozoa | 5 |
|  | 92193ad450481715e52e4a9a83cda3cc | Stramenopiles | Ochrophyta | 5 |
|  | 1ce3dd7f15e1df71eb14a591ffc4380d | Rhizaria | Cercozoa | 5 |
|  | e794ed4635366274ea74b1c1cc7c0044 | Alveolata | Ciliophora | 5 |
|  | 98236311617f66aae534137c028cd248 | Holozoa | Metazoa_(Animalia) | 5 |
|  | 1184851f155294c39953b76f44056338 | Nucletmycea | Fungi | 5 |
|  | f045ef6e2b3f2809629683c660bb7304 | Stramenopiles | Ochrophyta | 5 |
|  | 2ea8ea4f6671db6cc8dee3b2d179e727 | Holozoa | Metazoa_(Animalia) | 5 |
|  | 5f62e510ff5a6a0706c59a9e2fc312de | Stramenopiles | Labyrinthulomycetes | 5 |
|  | f2cb64963031eb545f12b3385ae28fc4 | Holozoa | Metazoa_(Animalia) | 5 |
|  | c7ccf0f1ee8654c4b183537cfb82f1ae | Nucletmycea | Fungi | 5 |
|  | 9f451f6580532598806c68e3b41af488 | Bacteroidia | Cyclobacteriaceae | 6 |
|  | c6d817be27dbc37ff5af4165f7fdd777 | Bacteroidia | NA | 6 |
|  | a9e933c5cab6ce67851a04e49d6f8319 | Verrucomicrobiae | Verrucomicrobiaceae | 6 |
|  | 7bf1f67e25cd6eee26198dca01db102c | Alphaproteobacteria | Rhodobacteraceae | 6 |
|  | b9aed3468734a89854a77ef226a74541 | Deltaproteobacteria | Oligoflexaceae | 6 |
|  | 8e3eb8afbe47abc8183a78fc49d15eb5 | Deltaproteobacteria | Oligoflexaceae | 6 |
|  | 70a7051bf87424b9471eee07087c293d | Verrucomicrobiae | Rubritaleaceae | 6 |
|  | 05817b544b5a93ec5fdad061d10ff44c | Bacteroidia | Saprospiraceae | 6 |
|  | 19ee0419de1a9ce5b21f798dbed21d77 | Deltaproteobacteria | Nannocystaceae | 6 |
|  | 59d1398136bd3482b233230a86982b44 | Melainabacteria | NA | 6 |
|  | 89878dd0c9f53eb2c31bfeef5bb214d4 | Planctomycetacia | Pirellulaceae | 6 |
|  | fef08367783b567eacf6d002aa9b6083 | Bacteroidia | Saprospiraceae | 6 |
|  | 42978440b1c4c8020a4a57553fa7b21a | Alphaproteobacteria | Rhodobacteraceae | 6 |
|  | 7f5bd1b5367f1b9285a3c3946cfdd136 | OM190 | uncultured_bacterium | 6 |
|  | 9dfeaf1904c0da6a9565bc0b692e9692 | Verrucomicrobiae | Rubritaleaceae | 6 |
|  | ff939ed5d3a632da4cc0e40118519169 | Bacteroidia | Flavobacteriaceae | 6 |
|  | b34be3d350600ff95dfa539e65468c79 | Alphaproteobacteria | Rhodobacteraceae | 6 |
|  | 2cf8b3a4ab8e087e656ff089fc682611 | Bacteroidia | NA | 6 |
|  | 67ed0bcf2555237d7ef361269b6faed6 | Bacteroidia | uncultured | 6 |
|  | 78aae98fb7a1f5e651b083e65a8c9a94 | Parcubacteria | uncultured_bacterium | 6 |
|  | 16d5135d061cee643a8e9e20011eca06 | Deltaproteobacteria | Nannocystaceae | 6 |
|  | e3be09b89e0ce90a4d7f78799461d156 | OM190 | uncultured_bacterium | 6 |
|  | 7b8bee811d5ea11d60af195b0f5395db | Deltaproteobacteria | Oligoflexaceae | 6 |
|  | 111d33a00573768ee72b8217b99eae8f | Verrucomicrobiae | Verrucomicrobiaceae | 6 |
|  | 893e5b3e560bafdbda6a31fd9f67d5ee | OM190 | uncultured_bacterium | 6 |
|  | b1c7b45c73f62d2c7c12546e952f24a2 | Chloroplastida | Chlorophyta | 6 |
|  | 4158bac92360eca4cf59ef32a7323a95 | Nucletmycea | Fungi | 6 |
|  | 46d25bd400b154ade0c3461dc36b41eb | Chloroplastida | Chlorophyta | 6 |
|  | 2d008e3f5ebc6c261b108531ae73b563 | Holozoa | Metazoa_(Animalia) | 6 |
|  | 816e8478a18610065b614dd87f87a8b1 | Nucletmycea | Fungi | 6 |
|  | a50ebba43fbd8f757b2d206c87b1a6ca | Stramenopiles | Ochrophyta | 6 |
|  | 61f772df3fe230b1fe23c175bb4f35fd | Stramenopiles | Labyrinthulomycetes | 6 |
|  | 795af489b506067e98457a563a941b9d | Alveolata | Ciliophora | 6 |
|  | f453e046493614d39cf6de77400dbe3a | Alveolata | Ciliophora | 6 |
|  | bac570b88f0c2c91cd008560a3283f50 | Stramenopiles | Ochrophyta | 6 |
|  | 3851302a834af48838624807e1e33a61 | Stramenopiles | Ochrophyta | 6 |
|  | 25ed45cea92e6fe822b436956c8259bc | Alveolata | Ciliophora | 6 |
|  | 230a830e0db0ca572dd254fb3b10075d | Stramenopiles | Ochrophyta | 6 |
|  | e34994b1b8b2335841498f2b07990401 | Alphaproteobacteria | Caulobacteraceae | 7 |
|  | 123e0039d8fe02799626467a8d4cfdf6 | Oxyphotobacteria | NA | 7 |
|  | 19ccd4a06f7d8ec8c926539cc89e7f6c | Alphaproteobacteria | Rhodobacteraceae | 7 |
|  | 306ed56293c852e3035bdacd6f324332 | Actinobacteria | Microbacteriaceae | 7 |
|  | a72c8d232576783fb4bf1d3740335cc0 | Alphaproteobacteria | Devosiaceae | 7 |
|  | eee5a11679039f975dc1d85b141f2dbb | Alphaproteobacteria | Rhodobacteraceae | 7 |
|  | 7c93a0e8725c8c126cede184fa0f27d7 | Bacteroidia | Cyclobacteriaceae | 7 |
|  | 13c65a299ba22da499d34b84d5ddbb02 | Alphaproteobacteria | Rhizobiaceae | 7 |
|  | f38372ad52fcd9de05fd209cea29919b | Alphaproteobacteria | Rhizobiaceae | 7 |
|  | 74e672d9a665fd5f1cef48dbc2e5e08e | Actinobacteria | Microbacteriaceae | 7 |
|  | 60c3f64ccf8a30afb8e7ab9fd246f4fb | Alphaproteobacteria | Rhodobacteraceae | 7 |
|  | adf76a6c70fb04a3b2e641d63f177930 | Bacteroidia | NA | 7 |
|  | 372f6cbb76f8f4921a1fedf7c243f810 | Alphaproteobacteria | Rhizobiaceae | 7 |
|  | 61e19093f78fcb34367de59e32286687 | Alphaproteobacteria | Rhodobacteraceae | 7 |
|  | b3ac5111ad7883ff986b59201637b4dc | Bacteroidia | NA | 7 |
|  | 197d33fa61383927266ef79914152b27 | Alphaproteobacteria | Rhodobacteraceae | 7 |
|  | c909debada5fd5696b38b92c45902b3e | Bacteroidia | NS11-12_marine_group | 7 |
|  | 04b131d6ae425287bb1cdb279649a8d4 | Phycisphaerae | Phycisphaeraceae | 7 |
|  | ff59438977738f7c4e88df13cb4f459f | Bacteroidia | Flavobacteriaceae | 7 |
|  | b4b3e0b0dac19419c23ffbb7d168cfd9 | Parcubacteria | uncultured_bacterium | 7 |
|  | 9749dd4d7c84cdb7682247e13fc9f4b9 | Stramenopiles | Ochrophyta | 7 |
|  | 419fae59d3521b3c5887fe298f300183 | Alveolata | Ciliophora | 7 |
|  | 3a2ae00ba178e208bef3c52a3c4c6251 | Alveolata | Ciliophora | 7 |
|  | ecb0cbf3a02d773958c19fcb528722ce | Alveolata | Ciliophora | 7 |
|  | fd365b4adf8198fa91654cf40c8601bc | Chloroplastida | Chlorophyta | 7 |
|  | e5efaa531aa5372c1b483652de8ced44 | Chloroplastida | Chlorophyta | 7 |
|  | 9db0f872e9c1f4cd7493522f6522fc73 | Chloroplastida | Chlorophyta | 7 |
|  | 103e073cf5354e2859959b748c0ec17c | Chloroplastida | Chlorophyta | 7 |
|  | 2ab28b44f494410eede35c92c3e46e52 | Stramenopiles | Ochrophyta | 7 |
|  | af06a3fd0cc6064b6c99a290aed5a8a9 | Stramenopiles | Ochrophyta | 7 |
|  | 34d9f0e2796eacc56418a883dab9e71f | Alveolata | Ciliophora | 7 |
| ASV_5 | a94ef47c814e7fcb8e3e7b084bdccd3b | Chloroplastida | Chlorophyta | 7 |
|  | b22dd0c778f3653ce0fc77dc45e0f7da | Stramenopiles | Ochrophyta | 7 |
| ASV_4 | 329a5b205df8a85fffc2cca9276eb50b | Chloroplastida | Chlorophyta | 7 |
|  | 41725c4d9ebbdd54a4fcf91498cda8c5 | Chloroplastida | Chlorophyta | 7 |
|  | ffb05732d5306df924e3dcb2fdafe1a4 | Stramenopiles | Ochrophyta | 7 |
|  | 508c1177024f11652f3a6c83bae9df38 | Verrucomicrobiae | Opitutaceae | 8 |
|  | 5417cce18ccd9269c3efcca59b07cfbd | Alphaproteobacteria | Sphingomonadaceae | 8 |
|  | 21fee0bc49eb1244ca1a0001ccb4875a | Planctomycetacia | Pirellulaceae | 8 |
|  | d272e0f74f1d80d085e1c52cb138afd9 | Alphaproteobacteria | Rhodobacteraceae | 8 |
|  | 2729c1fb426c8fab8015995b4d418d83 | Verrucomicrobiae | Rubritaleaceae | 8 |
|  | 325fada27537679829d71bd3b86ba0b6 | Alphaproteobacteria | Rhodobacteraceae | 8 |
|  | 2e76c1fa8acd5706fb5f5eae6bdfae23 | Bacteroidia | Flavobacteriaceae | 8 |
|  | 0250001bb0b6d22710c37ef671c96bfe | Alphaproteobacteria | Caulobacteraceae | 8 |
|  | 3c777616f927a4e2fd419630471545ef | Bacteroidia | Cyclobacteriaceae | 8 |
|  | a998588c03c3a069955703f91fac0503 | Bacteroidia | Spirosomaceae | 8 |
|  | 0a85a504fac34181b965c6af3321d4f7 | Alphaproteobacteria | Caulobacteraceae | 8 |
|  | 479fc8ab472f6b2734c2dc4922a686bd | Alphaproteobacteria | Devosiaceae | 8 |
|  | 4299f7d6a2e4fee1636c036e3613d781 | Holozoa | Metazoa_(Animalia) | 8 |
|  | fc3ab0deb43a7d0842d3b3d5f1942da0 | Rhizaria | Cercozoa | 8 |
|  | 5af29b53847a4c2b6d1a6518f8a9c639 | Holozoa | Metazoa_(Animalia) | 8 |
|  | be8e54d871b3b74e7e9d5dd4a3543d79 | Chloroplastida | Chlorophyta | 8 |
|  | ff778b6174ae6f2d3c81b007793789ed | Holozoa | Metazoa_(Animalia) | 8 |
|  | 068efb5f8ebd51b95a18d00f5bd362ea | Chloroplastida | Charophyta | 8 |
|  | 19c7fdd70bd8c3007ba0d893bbb229ba | Nucletmycea | Fungi | 8 |
|  | e69d4b857794aa81c4a4de76aa973cb2 | Nucletmycea | Fungi | 8 |
|  | 985ddbcf19d4aa3921ab8b973585f5ef | Nucletmycea | Fungi | 8 |
|  | 013d7dc057e0c69a9863725629eceb51 | Holozoa | Metazoa_(Animalia) | 8 |
|  | 72502e4bbb7645823bdf2bd513a6c897 | Holozoa | Choanoflagellida | 8 |
|  | 33a0dd89f08bbf29b4818db690a101b4 | Rhizaria | Cercozoa | 8 |
|  | b36a6d2de7efe48dea93b6d5ab877540 | Holozoa | Metazoa_(Animalia) | 8 |
|  | 857001cf625c79196a50338b47a3f01d | Holozoa | Metazoa_(Animalia) | 8 |
|  | 11c22da9dfbaea5f81908d4bece3e94f | Nucletmycea | Fungi | 8 |
|  | 27d5a1b793090ca68b9a12606c2ea35e | Discosea | Flabellinia | 8 |
|  | db32ebb1cd48d01a7b08dfc5cfc602fd | Alveolata | Ciliophora | 8 |
|  | a4b1cb962ffd4049e87bf40c224147d7 | Holozoa | Metazoa_(Animalia) | 8 |
|  | 65ff2a8242ffde42eb2cf63731f9f8b8 | Alveolata | Ciliophora | 8 |
|  | bd342f0ebbc7617161890cb4dd92713f | Rhizaria | Cercozoa | 8 |
|  | fcf2e16546e0eef7874b8524fbd659f8 | Alveolata | Ciliophora | 8 |
|  | 38a6f6c269bbd209d712253d35db3078 | Holozoa | Metazoa_(Animalia) | 8 |
|  | 9248881ff3c028be35c37ba4e40e012e | Alphaproteobacteria | Rhodobacteraceae | 9 |
|  | eae8ecbb7b9171d1fc7b098069c2d35d | Bacteroidia | Saprospiraceae | 9 |
|  | 5dfc8787e748c174a5760057d11a60b9 | Bacteroidia | Saprospiraceae | 9 |
|  | 3b0b672ec4cab21e2613fd4ea4c074f4 | Bacteroidia | Chitinophagaceae | 9 |
|  | e725d756936a6ab7738ef30f3ecf4761 | Verrucomicrobiae | Verrucomicrobiaceae | 9 |
|  | 3e45c1ff2cdf6fad19ddc5ca18d4c224 | Bacteroidia | NA | 9 |
|  | 2e566132604bc46202c0780a0e0d5f2b | Verrucomicrobiae | Verrucomicrobiaceae | 9 |
|  | 2d510da4815b9971fa554fdf4c5eaf4d | Bacteroidia | Cyclobacteriaceae | 9 |
|  | c6d06acd2529b730412b160cef41e1f0 | Alphaproteobacteria | Hyphomicrobiaceae | 9 |
|  | fba540739dea3bf315a8649bfe171a5f | Bacteroidia | Chitinophagaceae | 9 |
|  | 762533d8f0e784d6211f1d70819d04f0 | Parcubacteria | uncultured_bacterium | 9 |
|  | fc795982a1e00e77015b1da9c3c4635b | Bacteroidia | Flavobacteriaceae | 9 |
|  | 6c51f19a82b3fe75a7275d6a034200bc | Bacteroidia | Saprospiraceae | 9 |
|  | 3e0b8cfb373c47252fab34907c74b7c4 | Alphaproteobacteria | Hyphomonadaceae | 9 |
|  | 89240be096298f4dcd18e73b914c7e61 | Verrucomicrobiae | Opitutaceae | 9 |
|  | 4e81690705a3153900bb5e0dcb6bc399 | Gammaproteobacteria | Alteromonadaceae | 9 |
|  | 089ef18b4e0f8aba246ad0d6d28cc673 | Bacteroidia | Flavobacteriaceae | 9 |
|  | 8d2596f498ac3bdac2f1c99f6c9bd41b | Verrucomicrobiae | Opitutaceae | 9 |
|  | 2f84a579ea82d59042fd863c46568d87 | Bacteroidia | Flavobacteriaceae | 9 |
|  | b4e7e41f1c5bb073369dad29a4350859 | Verrucomicrobiae | Rubritaleaceae | 9 |
|  | 00c9bb9b755d00d2185a4bcc89cd1d27 | Bacteroidia | Flavobacteriaceae | 9 |
|  | d334302432e2b535e0e93cb78977b289 | Alphaproteobacteria | Beijerinckiaceae | 9 |
|  | 2c5667dc183e672562d086f88967379a | Parcubacteria | uncultured_organism | 9 |
|  | cc76e49a01c59f967d48374d75dafc3a | Verrucomicrobiae | Pedosphaeraceae | 9 |
|  | 8afa576ad00fab3a00de0b3f0f327205 | Bacteroidia | Saprospiraceae | 9 |
|  | a39369fde7c7f4687f5ed5f2b166a163 | Verrucomicrobiae | Rubritaleaceae | 9 |
|  | 03cbb9ded16c104751628a75747079b3 | Nucletmycea | Fungi | 9 |
|  | d1244949cf660afe33bf445802a4a590 | Nucletmycea | Fungi | 9 |
|  | 448c9deb2ed17e9f8fe88e88b5fc2d0d | Alveolata | Ciliophora | 9 |
|  | 25290ac4898369a32c9b8620a4eae81b | Chloroplastida | Chlorophyta | 9 |
|  | 174bdae7efd598425fde827ec2319bca | Stramenopiles | Ochrophyta | 9 |
|  | 8c2ab4a6a06748ef2b78b2e0583cfb20 | Nucletmycea | Fungi | 9 |
|  | 2d8811a6c6b02875a36afc63df34f744 | Nucletmycea | Fungi | 9 |
|  | 732d17fd96b98b99c746f5a72031936f | Alphaproteobacteria | Sphingomonadaceae | 10 |
|  | 9e1f06330a2fbfd106deef0803d1b676 | Bacteroidia | Saprospiraceae | 10 |
|  | cee046516c280920b47da0461193380c | Oxyphotobacteria | Pseudanabaenaceae | 10 |
|  | 47e6a1ba6d8a2a6db1b306edeedb1575 | Bacteroidia | Saprospiraceae | 10 |
|  | 4a78bafd443dd4bd1ce483a9b7cf9b64 | Bacteroidia | Saprospiraceae | 10 |
|  | 24163c2595447dd869e119dfe9fb4282 | Bacteroidia | Saprospiraceae | 10 |
|  | 2c218a5ce3fdc36665ff7c2f02039858 | Verrucomicrobiae | Pedosphaeraceae | 10 |
|  | a1429a93959f021265543a4d072ebfb4 | Bacteroidia | Saprospiraceae | 10 |
|  | ee0cd8e421bbc4aec532b4beed18f71b | Alphaproteobacteria | Rhizobiaceae | 10 |
|  | 05356b79dcdc4d48a1dab4681ffe2338 | Verrucomicrobiae | Rubritaleaceae | 10 |
|  | 7e27a856c5d2b8086229da0ea9846aa5 | Alphaproteobacteria | Rhizobiaceae | 10 |
|  | 8711020a7c5b0e61137018885c56305e | Gammaproteobacteria | Burkholderiaceae | 10 |
|  | cb87c562e53907f3efa52fb3fe5013a7 | Planctomycetacia | Pirellulaceae | 10 |
|  | 8f00c01efa61fccba3bc48c93cfafb8f | Melainabacteria | uncultured_bacterium | 10 |
|  | 870d055d9ade888514fa30f8a11f8d9d | Chloroplastida | Chlorophyta | 10 |
|  | dafdcd781c461e6ec601d589d3ced050 | Rhizaria | Cercozoa | 10 |
|  | ef5fe51fabdcf2066b338398694f5836 | Chloroplastida | Chlorophyta | 10 |
|  | 6aa49f5d67527b5fa9f23f04e89c419a | Nucletmycea | Fungi | 10 |
|  | a871bf94b6a91d8b44e9a183e4f39059 | Holozoa | Metazoa_(Animalia) | 10 |
|  | 720d5d6e4b66f011740f06e9ee440ce7 | Chloroplastida | Chlorophyta | 10 |
|  | ffd45d058a93a02dca611c07a29b68c0 | Nucletmycea | Fungi | 10 |
|  | 00f6f834cf4888fae5f4e7d360432bab | Holozoa | Choanoflagellida | 10 |
|  | 3eb02a5be9437222db737f98a71628c7 | Stramenopiles | Ochrophyta | 10 |
|  | c01d0a3b4fccd4787a121eb61468a835 | Tubulinea | Euamoebida | 10 |
|  | 267d3fabd1cc150401e3c6e5dc37783a | Nucletmycea | Fungi | 10 |
|  | 2034fa584bda2e2bbb624b9eaf3fb8bc | Alveolata | Ciliophora | 10 |
|  | 45ffba4a465258f6296c1a2d11e16073 | Chloroplastida | Chlorophyta | 10 |
|  | 354b2cc13cc16a29f98a1a247e5e956a | Stramenopiles | Ochrophyta | 10 |
|  | 58236806e88cc9af4b0c6495022db87b | Alveolata | Ciliophora | 10 |
|  | 2d44d6b42920919042b2a43e34f1d64b | Alveolata | Ciliophora | 10 |
|  | 3603183408e581618c222c86343b4c8e | Stramenopiles | Ochrophyta | 10 |
|  | 211e5b31dac6ff8079d53eb629c6d970 | Nucletmycea | Fungi | 10 |
|  | 05aee6c24636a9d8273a772482c755a1 | Alveolata | Ciliophora | 10 |
|  | 34566c79359017af2c71fa691d59876f | Bacteroidia | Flavobacteriaceae | 11 |
|  | ba758c974e3acd5b80adec56eeb36bf0 | Alphaproteobacteria | Sphingomonadaceae | 11 |
|  | 669c717d944f89b7f26b3bd5513ca5c4 | Deltaproteobacteria | Oligoflexaceae | 11 |
|  | 426f5d31606c56fac9187546f8d8444a | Alphaproteobacteria | Geminicoccaceae | 11 |
|  | 55e41785edb5cb0202a57f3ff832c8da | Alphaproteobacteria | Micavibrionaceae | 11 |
|  | 1d51d96d8ee483065ca6e51b24694211 | Deltaproteobacteria | Oligoflexaceae | 11 |
|  | 45eb850e1abb425784be3c3b56974781 | Bacteroidia | NA | 11 |
|  | 9ea7cda26692a80993be992099605220 | Alphaproteobacteria | Rhizobiaceae | 11 |
|  | 81546166f8cc36ba21b93b3a71a1e980 | Gammaproteobacteria | Burkholderiaceae | 11 |
|  | e7fda335ab98974fc507e35218aa2fbb | Gammaproteobacteria | Legionellaceae | 11 |
|  | a904fc3381c14956cfea87b327b3d986 | Discoba | Discicristata | 11 |
|  | 548cfba8f06767ad01ead95e801bf597 | Alveolata | Ciliophora | 11 |
|  | f70a43ef9501603168fe8a34a70c9238 | Nucletmycea | Fungi | 11 |
|  | 1f49b5b3772432da13b85a91fabe7357 | Nucletmycea | Fungi | 11 |
|  | aa38a4ebebf493e0c1d4030a5bb76a32 | Rhizaria | Cercozoa | 11 |
|  | 5857c383522a6e585e8ff73aab81dd61 | Alveolata | Ciliophora | 11 |
|  | 4cfccf6a1185d8c9a190e2558685bd7d | Alveolata | Ciliophora | 11 |
|  | 645ce7d8fd2152025e51daed0c3be6a6 | Stramenopiles | Ochrophyta | 11 |
|  | 6a11d595a61d336726467bd1620f0bb1 | Stramenopiles | Ochrophyta | 11 |
|  | aeee3c42da68de6ab626541bfb745d0d | Stramenopiles | Ochrophyta | 11 |
|  | a66c0c17fa0f24891e53feb101e2dc6b | Alveolata | Ciliophora | 11 |
|  | a3c543a5b5e3d7b6a49333dad46d6e44 | Stramenopiles | Ochrophyta | 11 |
|  | 3bde682f0c31d0178f968db0e65f161b | Stramenopiles | Ochrophyta | 11 |
|  | a2f527e94fa1c57e64ed370b6524a1cc | Nucletmycea | Fungi | 11 |
|  | 460d0ed068eafaf78eade351880d1f07 | Stramenopiles | Ochrophyta | 11 |
|  | 30fb7e06a12ee39dd71b2a086df8e89c | Stramenopiles | Ochrophyta | 11 |
|  | 13242cba88c1ce62da6278c3c612b1cd | Holozoa | Metazoa_(Animalia) | 11 |
|  | 16a6049710836ea77e481801abff5547 | Bacteroidia | Saprospiraceae | 12 |
|  | 8714fc6dc77842d41d9f9b3da1bd9b19 | Alphaproteobacteria | Rickettsiaceae | 12 |
|  | 1281f67ca783d43193883aa10c35119e | Alphaproteobacteria | Devosiaceae | 12 |
|  | 47e52b9642d26df59187e793cfcaa525 | Deltaproteobacteria | NA | 12 |
|  | adfeb5223a069149cbacf2a814a4df27 | Actinobacteria | Mycobacteriaceae | 12 |
|  | 4ed15c0cdfdac940669af07f83ac6114 | Planctomycetacia | Pirellulaceae | 12 |
|  | 82e1463c40ae1127e73575ad0cfdb5aa | Alphaproteobacteria | Geminicoccaceae | 12 |
|  | bb4fdacf263f0c663b25844c729b52c4 | Planctomycetacia | Pirellulaceae | 12 |
|  | b42899f880c5afafce05bd72f46ed945 | Bacteroidia | NA | 12 |
|  | d1cb8c1044f1d0913e9b5e8cc961ede1 | Alphaproteobacteria | Rhodobacteraceae | 12 |
|  | b5380cbe1ca1f0b0954bd06b3ed67931 | Oxyphotobacteria | NA | 12 |
|  | 6c62b4dfdc11866c4db332efb0df2601 | Bacteroidia | NA | 12 |
|  | b73f7eb1105b774fdd1bfed9171f364d | Rhodothermia | Balneolaceae | 12 |
|  | 81a921c97270c29dd70ce1dc3c3fdb3b | Planctomycetacia | Pirellulaceae | 12 |
|  | 5e6c53bb955cad75f4e4e3b515f518bc | Parcubacteria | NA | 12 |
|  | c76d5770bd7bf0920479a897d06022d3 | Alphaproteobacteria | Rhizobiaceae | 12 |
|  | 15be657a9506f4d98a9b53277f6af0ef | Bacteroidia | NA | 12 |
|  | 3a04fc40426b7986e6290f8136fd598b | Stramenopiles | Ochrophyta | 12 |
|  | 2eaf29be26c07dc9bb4ca0e31855c136 | Holozoa | Metazoa_(Animalia) | 12 |
|  | 9d1e8685a4ca6e80d4f92e88331f4c40 | Stramenopiles | Ochrophyta | 12 |
|  | b54c010cc10a19b4d79e9cac09e458c5 | Stramenopiles | Labyrinthulomycetes | 12 |
|  | 5d257b421471c2fac361fe1682c8943f | Alveolata | Ciliophora | 12 |
|  | d5037075fc48479607524f0b2f47822c | Nucletmycea | Fungi | 12 |
|  | f4b737082a54197f55d764b1ecd8615e | Stramenopiles | Ochrophyta | 12 |
|  | 260ef3484460069aa594679cbf32c9fb | Alveolata | Ciliophora | 12 |
|  | ba95dbbcf28ca0f992ba686acd83c9d3 | Alphaproteobacteria | Rhodobacteraceae | 13 |
|  | 293caa46ffacba2ee84f71278dcd1cff | Alphaproteobacteria | Devosiaceae | 13 |
|  | 6f81caba070f50b663e9c7fac16f634e | Alphaproteobacteria | Micavibrionaceae | 13 |
|  | d702e08ecc99786d97278ad1cda9cad1 | Bacteroidia | Saprospiraceae | 13 |
|  | c4f79f6cc9acf833a162bfb46fccb06d | Alphaproteobacteria | Rhodobacteraceae | 13 |
|  | 5a476e4041aea093b24b11aee3e12462 | Deltaproteobacteria | Bdellovibrionaceae | 13 |
|  | d7f03e29a5d907e2594a9cd0ba71cfd3 | Bacteroidia | Cyclobacteriaceae | 13 |
|  | 2e37032e8ba339bc38fb4ba5c2080a43 | Bacteroidia | Cryomorphaceae | 13 |
|  | 1aad8b6785b535c38ada22d60620d910 | Bacteroidia | Saprospiraceae | 13 |
|  | 7ea1642bcc58abdb08e054c83d02e00d | Alphaproteobacteria | Rhizobiaceae | 13 |
|  | e95c38c7663f65248bcd99c235a246ee | Alphaproteobacteria | Devosiaceae | 13 |
|  | d291d578fd9a6cb419de6a65cea82fb6 | Planctomycetacia | Pirellulaceae | 13 |
|  | 4ce1227352e7511a6673843b9dc355d3 | Bacteroidia | NS9_marine_group | 13 |
|  | 73deecfdba2721a8d74a9516a3d1a924 | Deltaproteobacteria | Bacteriovoracaceae | 13 |
|  | 7367a2f6cd2a3fe09b52ba477ff4c89e | Alphaproteobacteria | Rhodobacteraceae | 13 |
|  | d52212aaa0ef5334765cb67531154133 | Holozoa | Metazoa_(Animalia) | 13 |
|  | dcdaa6dad3dd11dd87702a3342c72caf | Holozoa | Choanoflagellida | 13 |
|  | 64df344db65692f418f288063b0b7440 | Nucletmycea | Fungi | 13 |
|  | 781c9ee04ad5264dc2c77c29d6a1d2f9 | Holozoa | Choanoflagellida | 13 |
|  | f41e7a5a6fc7522c2a3dc5637e5a5bf9 | Holozoa | Choanoflagellida | 13 |
|  | 88e2249fe83bbe1567a5a4c765cbbb8a | Stramenopiles | Labyrinthulomycetes | 13 |
|  | 61f191e8d8d6dc9f30fc4de4c404baeb | Stramenopiles | Ochrophyta | 13 |
|  | acd7634a2720a6368d138e3559dd2f93 | Stramenopiles | Labyrinthulomycetes | 13 |
|  | 4cae2618fd003b48d6c71b6320b16dab | Discoba | Discicristata | 13 |
|  | e6bb0d76d6ce4207dc4a5953bacd383e | Verrucomicrobiae | DEV007 | 14 |
|  | 0fd4d6b8d734e0cc0412a7e22580b6c0 | Alphaproteobacteria | Rhodobacteraceae | 14 |
|  | d69a47c729d16c75dfc4c453e343d4bb | Anaerolineae | Caldilineaceae | 14 |
|  | 5e2666737e2d629068045bfdc8d941a5 | Bacteroidia | Saprospiraceae | 14 |
|  | 8cbe28e9d4071e2f72f8106725d8bde8 | Gammaproteobacteria | NA | 14 |
|  | 8ace7dc514808ec270f99fe93dcd3b80 | Bacteroidia | NA | 14 |
|  | f2e2d9016173d52f734aaa59721d8656 | Gammaproteobacteria | Xanthomonadaceae | 14 |
|  | f9bbd6048cbf51c81edb6cff452d1520 | Bacteroidia | NA | 14 |
|  | f1b65ee76a34ff76f1780a6f26b6a067 | Parcubacteria | uncultured_bacterium | 14 |
|  | 8c06bd8212a5dc4f7faa7699ab3f0d36 | Ignavibacteria | NA | 14 |
|  | 204b2603663634ea4750a5cd52c156d3 | Blastocatellia_(Subgroup_4) | Blastocatellaceae | 14 |
|  | 684df4e3f625aeea2b490a395dc2b7b6 | Gammaproteobacteria | Francisellaceae | 14 |
|  | 7f0e149358b9d64a07ba10af2863dada | Parcubacteria | NA | 14 |
|  | 6a441765cecc413bccbaf8c33ff86648 | Planctomycetacia | Pirellulaceae | 14 |
|  | f722ef9d9d15408f104eccd43db2fa1e | Holozoa | Choanoflagellida | 14 |
|  | 5b4be387696200465fc9994f7ca0b158 | Chloroplastida | Chlorophyta | 14 |
|  | b2e4f6932bc22960dc2b1da8878dde9a | Nucletmycea | Fungi | 14 |
|  | 013712f2276ae88c241cfb29d50ef603 | Stramenopiles | Ochrophyta | 14 |
|  | 6ea677ebfd240cbb4cf47cf6c71ca359 | Stramenopiles | Ochrophyta | 14 |
|  | aa2771105357653082f1a23ded6d8ecf | Chloroplastida | Chlorophyta | 14 |
|  | da5979041ccfe80cf31f04afb384fcfb | Alveolata | Ciliophora | 14 |
|  | 12d97a6a93fa128ffcc7d51374b886d1 | Rhodophyceae | Porphyridiophyceae | 14 |
|  | c43126d5c8fcf490b78b147e265b7340 | Alveolata | Ciliophora | 14 |
|  | 19ae794ab1554ceaf0b09414bd17431c | Stramenopiles | Ochrophyta | 14 |
|  | 29dcb17e1873ccf18e5b0df2b973e3b9 | Alphaproteobacteria | Rhizobiaceae | 15 |
|  | 2c9dc59e7894b3eb77c38eb1f9283cdb | Alphaproteobacteria | NA | 15 |
|  | 7de644ca4f747eb91dd38b9fbb5d1e52 | Alphaproteobacteria | Devosiaceae | 15 |
|  | 2918ee255dcc41b60d162542578a6f2d | Gammaproteobacteria | Burkholderiaceae | 15 |
|  | 03431dd783faf9d5585e94f769040d9a | Alphaproteobacteria | Micavibrionaceae | 15 |
|  | 3eb91aceb70082f6f8aba8c02f8fdccc | Bacteroidia | Flavobacteriaceae | 15 |
|  | 1d81a45db6eabb78fc13b891f993bb32 | Alphaproteobacteria | Micavibrionaceae | 15 |
|  | 054c737405cc9d5cd0563eff2854cb1a | Alphaproteobacteria | Micavibrionaceae | 15 |
|  | c3fac900573ea9fb77224a5b983bff79 | Alphaproteobacteria | NA | 15 |
|  | 1902d08f48735f05c4c5b0f744b98f10 | Bacteroidia | Spirosomaceae | 15 |
|  | f8f583b9c837ef032f1a44734f8730f4 | Bacteroidia | Flavobacteriaceae | 15 |
|  | ad229876eafabb367ca5f74786d90f44 | Alphaproteobacteria | Micavibrionaceae | 15 |
|  | 3f512bee1a3051cd427f43c037c54265 | Bacteroidia | Spirosomaceae | 15 |
|  | a275dda97d3c43bde610da9a2f618ca1 | Chloroplastida | Chlorophyta | 15 |
|  | feafbed2970faecf8e358a9e04fe3740 | Alphaproteobacteria | Rhizobiaceae | 16 |
|  | c38ceed6c4c4ed5c5442e8e63f593af2 | Alphaproteobacteria | Rhizobiaceae | 16 |
|  | 89ee65177c5b0b6d1811120ab7a80ab4 | Gammaproteobacteria | Legionellaceae | 16 |
|  | 8c1550347504b12f877f54d118eb1612 | Bacteroidia | Flavobacteriaceae | 16 |
|  | 0b512321243ac91ad53448ce19c08e7a | Bacteroidia | Flavobacteriaceae | 16 |
|  | 2f1f3e41baea9284d968ed6779e52c78 | Deinococci | Trueperaceae | 16 |
|  | 084f0dbe00c143303149e94dccb9e0ab | Planctomycetacia | Pirellulaceae | 16 |
|  | 8ed3be657c64dbe8d7b207ced75ee2da | Bacteroidia | Saprospiraceae | 16 |
|  | 2dbacd1685bd231d866a96ec0ad7e56b | Deltaproteobacteria | Bdellovibrionaceae | 16 |
|  | 71f1c792b209ff6e5a563d20954deb21 | Stramenopiles | Ochrophyta | 16 |
|  | c98877bb9ad89ac62a60ec2aef003c6b | Stramenopiles | Bicosoecida | 16 |
|  | 455b608c989a530966a45a733af46160 | Nucletmycea | Fungi | 16 |
|  | f53b47d2c2d60f9f39779cbac2004900 | Alveolata | Ciliophora | 16 |
|  | ea5535818b58fd22d79023d8ba6b4fe5 | Nucletmycea | Fungi | 16 |
